# Homodimeric and Heterodimeric Interactions among Vertebrate Basic Helix–Loop–Helix Transcription Factors

**DOI:** 10.3390/ijms222312855

**Published:** 2021-11-28

**Authors:** Ana Lilia Torres-Machorro

**Affiliations:** Laboratorio de Biología Celular, Departamento de Investigación en Fibrosis Pulmonar, Instituto Nacional de Enfermedades Respiratorias “Ismael Cosío Villegas”, Tlalpan, Mexico City 14080, Mexico; ana.torres@iner.gob.mx; Tel.: +52-(55)-54871700 (ext. 5257)

**Keywords:** transcriptional regulation, E-proteins, ID proteins, sequence-specific transcription factors, DNA binding, Class II bHLH transcription factors, protein–protein interactions

## Abstract

The basic helix–loop–helix transcription factor (bHLH TF) family is involved in tissue development, cell differentiation, and disease. These factors have transcriptionally positive, negative, and inactive functions by combining dimeric interactions among family members. The best known bHLH TFs are the E-protein homodimers and heterodimers with the tissue-specific TFs or ID proteins. These cooperative and dynamic interactions result in a complex transcriptional network that helps define the cell’s fate. Here, the reported dimeric interactions of 67 vertebrate bHLH TFs with other family members are summarized in tables, including specifications of the experimental techniques that defined the dimers. The compilation of these extensive data underscores homodimers of tissue-specific bHLH TFs as a central part of the bHLH regulatory network, with relevant positive and negative transcriptional regulatory roles. Furthermore, some sequence-specific TFs can also form transcriptionally inactive heterodimers with each other. The function, classification, and developmental role for all vertebrate bHLH TFs in four major classes are detailed.

## 1. Introduction

Transcription factors (TFs) are proteins that are directly involved in the activation or repression of RNA synthesis from a DNA template [1], most of the time by recognizing specific DNA sequences [2]. Thus, a set of related sequences preferred by a given transcription factor are known as the TFs’ DNA-binding motifs [2].

TFs can be broadly classified as either basal (general) or sequence-specific TFs [3]. The general TFs recognize the core promoter and are directly involved in RNA polymerase recruitment and transcription initiation. In contrast, the sequence-specific TFs regulate transcription initiation at specific promoters by identifying precise DNA motifs located in enhancers. These enhancers can be proximal or distal to the core promoter [3]. The signaling between the sequence-specific transcription factors and the core machinery is mediated by co-activators and co-repressors [3].

The sequence-specific DNA-binding TFs have also been classified based on their well-defined DNA-binding protein domains [4]. These TFs families include the basic helix–loop–helix (bHLH), C2H2 zinc finger (ZF), homeodomain, and basic leucine zipper (LZ) groups (reviewed in [2]).

TFs usually cooperate and synergize with other TFs through extensive protein–protein interactions within their TF family and different families [5]. This combinatorial TF structure provides precision and flexibility to the transcriptional program operating in diverse cell types and tissues [5]. However, a detailed view of how specialized transcriptional networks function is still an emerging research field, despite the enormous progress.

The latest, 2019, bHLH TF family comprehensive review [6] summarized their role in various regulatory pathways. However, in most cases, it did not detail the dimeric form involved. This work aims to summarize the complexity of the vertebrate bHLH TFs network, recapitulating all protein–protein interactions known among E-protein interacting bHLH TFs. In addition, detailed information about the experimental approaches defining these interactions is included. Tissue-specific TFs dimers with E-proteins are extensively known. The new conceptual insight from this review highlights the regulatory relevance of tissue-specific dimers not involving E-proteins, reshaping the information summarized before [6] and expanding the classical model. 

## 2. The bHLH TF Family

**Figure 1:**
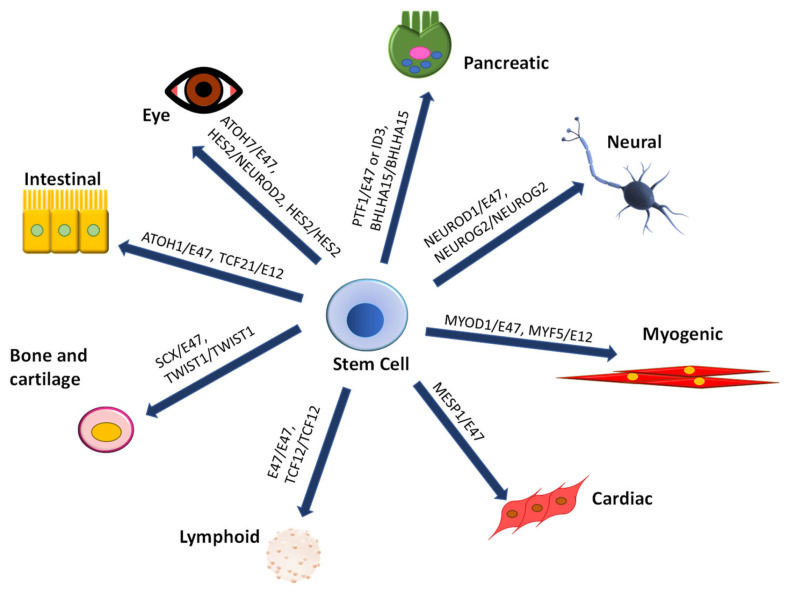
Developmental involvement of bHLH TFs. The diagram shows some developmental pathways regulated in part by bHLH TFs. Examples of specific dimeric bHLH TFs forms involved in differentiation and tissue/cell development are shown. Protein dimers are written as monomers separated by a diagonal. The bHLH TFs also have a solid contribution to disease, which is thoroughly reviewed elsewhere [14,15,16,17].

The bHLH family is one of the most prominent among transcription factors [1,5] and is involved in cell differentiation and tissue development [7,8] (Figure 1). Table S1 lists the developmental involvement of the TFs summarized in this review. These TFs share a characteristic protein structure composed of a basic region [9] that interacts with DNA and a neighboring helix–loop–helix region that mediates dimerization [10]. Most bHLH dimers recognize the E-box, a hexameric sequence in the DNA with the consensus sequence CANNTG [11]. Nevertheless, further characterization and classification of the bHLH TFs groups revealed that some bHLH TFs could also recognize alternative sequences such as the N-box and the ESE-box [12,13]. These bHLH TFs modulate gene expression through dimer formation, combining activators or repressors with ubiquitous proteins (E-proteins) [7,8]. 

## 3. Classifications of the bHLH TFs

Back in the 1990s, the bHLH TFs from *Drosophila* and mammals were initially classified into three groups: Class A, B, and ID [18]. Class A corresponded to proteins expressed in all tissues tested and included E12 and E47. MyoD belonged to the Class B group characterized by TFs expressed only in some tissues as heterodimers [19]. The ID class opposed the action of A and B TFs [20].

With the burgeoning number of bHLH TFs being identified, several other classification schemes have emerged to accommodate our growing understanding. The Murre Lab initially classified the bHLH TFs into six classes (I–VI, Table 1) based upon dimerization capabilities, DNA-binding specificities, and tissue distribution [6,21,22]. The Class I group, or E-proteins, were expressed in many tissues [23] and could participate in homo- and heterodimers. E-proteins were initially considered to have redundant roles with other E-proteins [24,25]. However, dimerization partners usually have a preferred E-protein [25,26,27,28,29,30,31,32,33,34,35]. MYOD1, NEUROD1, and SCX belonged to the Class II group, characterized by tissue-specific expression and heterodimerization with E-proteins. Class II was extended in 2002 when bHLH TFs were reviewed again, and new TFs were added after identification with a computational approach [36]. Class III TFs had an additional leucine zipper (LZ) motif, with the TF MYC as an example. Proteins heterodimerizing with class III TFs (MAD, MAX, MXI) belonged to the Class IV group. The ID proteins, which lacked the basic domain and interacted with Class I and II proteins to repress their function, constitute the Class V group. The Class VI TFs were homologous to *Drosophila*’s bHLH TFs hairy and enhancer of split and generally functioned as repressors (e.g., HES1, HEY2). Later, Class VII was added to accommodate the PAS-domain proteins [6].

Early on, the bHLH TFs were also classified as groups A to D [42] based on phylogenetic sequence comparison of the bHLH motif and the DNA-binding specificity. Each of the four groups recognized a specific E-box sequence in the DNA. Group A included E-proteins and the TFs of Class II, defined above. Group B was composed of Murre’s TFs Classes III, IV, and VI and could be further subdivided due to an LZ motif’s presence or absence. PAS-domain proteins belonged to the C group, and ID proteins went into the D group.

Ledent and collaborators [43] expanded the Atchley and Fitch [42] classification above with two groups: The E group, now containing the Murre’s Class VI proteins, and the F group, which had an additional COE (Collier/Olf1/EBF) domain [43,44,45]. Moreover, numerous bHLH motifs from other organisms such as *C. elegans* and mouse were included in the phylogenetic analyses. These additions resulted in further classification of the bHLH TFs into orthology families [44], where the Atchley’s D group became members of the A group. Some classifications kept D TFs as an independent group [46].

Afterward, the complete amino acid sequences of the bHLH TFs of seven different species (human, mouse, rat, worm, fly, yeast, and plant) were used to carry out phylogenetic analyses that identified six new clades [47]. Clades 1 to 5 contained bHLH genes formerly classified as Classes I and II [22]. Clade 1 was made up primarily of mammalian genes previously considered to belong to Class II. Clade 2 included previous Classes I, II, and V. Whereas Clade 3 contained myogenic factors and some previous group II proteins. Clade 4 concentrated on proteins with an additional LZ region, and some Clade 5 members contained genes with PAS domains. Clade 6 was specific to plant genes [47]. This analysis developed a phylogenetically precise relationship among bHLH genes and a new nomenclature based on the clade distribution [48]. 

Table 2 categorizes each factor according to the three bHLH TF classifications detailed above. As this review focuses only on vertebrates, a list of source model organisms for data summarized for each TF is included. Table 2 also lists each factor’s general transcriptional regulatory function, derived predominantly from transcriptional reporter assays. Caution should be taken when analyzing this information as unnoticed heterodimerization could be promoting context- or cell-dependent functionality [49,50,51].

## 4. Dynamic Nature of the bHLH TFs

The bHLH TFs function cooperatively as homodimers (E47/E47), heterodimers (MYOD1/E47), trimers (TAL1/E47/LIM), or multimeric structures [107,124,127,177,178]. These protein–protein interactions within the bHLH family are highly dynamic and cell and context dependent [51]. This cooperativity impacts the function, DNA-binding preferences, cofactor interactions, subcellular localization, and interactions with other proteins [179,180]. Thus, the developmental fate of each cell and tissue is related to the composition of the functional bHLH dimers or multimers present [5,66,181].

Dimeric interactions within the bHLH TF family are summarized in Table 3 (parts A and B), Table 4 (parts A and B), and Table 5, specifically among bHLH protein classes capable of interacting with E-proteins. These TFs belong to Classes I, II, V, and VI as grouped by the Murre Lab. TFs in groups III and IV were not included as their transcriptional network has been recently reviewed [37]. The bHLH TFs also participate in interactions with TFs from other families, including, for example, the LIM-domain protein family, excellently reviewed elsewhere [182]. 

In many cases, phosphorylation of the bHLH TF regulates its dimerization. NEUROG2 homodimers are efficient transactivators. However, when NEUROG2 is phosphorylated, it heterodimerizes with E47, reducing its transactivator capacity [31]. For OLIG2, its phosphorylation promotes homodimerization and transcriptional repression. OLIG2 dephosphorylation promotes heterodimerization with NEUROG2, a relevant process required for the motor neuron-oligodendrocyte fate switch [148]. Table S1 provides references for bHLH factors known to be regulated by phosphorylation. 

## 5. The Current Functional bHLH Model

The Murre classification scheme (Table 1) was selected for this review because it separates E-proteins from tissue-specific TFs and classifies ID proteins and the HES family in independent groups. Publications by the Murre Lab proposed a general way in which bHLH TFs function and interact. This model is widely accepted by previous and current publications in the field [14,25,62,129,138]. Briefly, Class I proteins were usually transactivators as homodimers or heterodimers with Class II, tissue-specific proteins. Class V proteins repressed many Classes I and II proteins, primarily by sequestering E-proteins, and Class VI proteins were transcriptional repressors [22].

Predictions of the functionality of bHLH TFs could be made based on the classification above and the other phylogenetic classifications; however, this could be misleading as each bHLH dimer’s function depends on the protein–protein interactions established. Thus, the relevance of this review originates from the need to summarize experimentally corroborated dimeric interactions among this TF family.

From Table 2, the following can be concluded: E-proteins are indeed transactivators as homodimers. However, E47 and E2-2 have also been reported to be context-dependent repressors. On the other hand, of 48 Class II TFs analyzed, 10 are only reported as transactivators, 12 only as repressors, 21 as both transactivators and repressors (or transcriptionally inactive dimers), and 5 remain untested. Furthermore, the majority of the Class II TFs can dimerize with E-proteins (Table 3, parts A and B). Nevertheless, this interaction with E-proteins does not always result in transactivation, as 19 class II TFs can sequester E-proteins in transcriptionally inactive dimers, and factors such as TCF21 and NEUROG3 can repress transcription as DNA-binding heterodimers with Class I proteins (Table 2). Likewise, some tissue-specific TFs can heterodimerize with bHLH TFs other than E-proteins (Table 4) or form homodimers (Table 5) with positive and negative transcriptional effects (see below).

## 6. bHLH Dimeric Interactions: The Importance of the Experimental Approach

Diverse experimental approaches, including in vivo and in vitro assays, have defined the dimeric interactions of bHLH transcription factors. Appendix A briefly describes these assays, classifying them as biochemical, biophysical, or genetic. 

The most common in vitro assay for testing dimeric bHLH interactions in the presence of DNA is the electrophoretic mobility shift assay (EMSA). This approach has been used since the discovery of the bHLH TFs and has demonstrated most E-protein homodimeric and heterodimeric interactions with Class II bHLH TFs. These interactions include all DNA-binding myogenic and neurogenic bHLH heterodimers. A major drawback of EMSA is that it cannot detect DNA-independent interactions or dimers that bind non-consensus or untested DNA sequences. Thus, unless a broader repertoire of DNA sequences was tested, such as in the CASTing assay [258], the possibility of interaction with another sequence (e.g., ESE-box) cannot be eliminated. Furthermore, a negative result in the EMSA only indicates that the dimer may not be binding to the DNA, as was the case for ASCL3 homodimers [119]. 

Some bHLH TFs dimers were discovered by alternate in vitro approaches, including GST-pulldown (GST), methylation interference footprinting (MIF), co-immunoprecipitation (coIP, also considered an ex vivo assay, Appendix A), X-ray mass spectrometry (MS), and circular dichroism (CD). The most common in vivo approach is the yeast two-hybrid (Y2H) assay, which has defined multiple E-protein dimeric interactions. Other in vivo approaches include the mammalian two-hybrid (M2H), the site-specific photocrosslinking (SSPC), and the fluorescence resonance energy transfer (FRET) assays. Excellent reviews elsewhere state the advantages and drawbacks of diverse protein–protein interaction methodologies [259,260]. 

MYOD and HAND1 are members of the select group of TFs that have confirmed dimeric interactions through multiple independent techniques, including EMSAs, coIPs, X-ray MS, and CD (Table 3, Table 4 and Table 5). TFs whose dimeric interactions have been verified using in vivo and in vitro assays are color-coded in yellow in the tables. 

The opposite situation is observed for MESP1, a TF whose dimeric interactions have only been analyzed with a single experimental technique, the Y2H. Even though most bHLH TFs have at least one verified interaction partner, a varied and complementary repertoire of experiments confirming dimeric interactions is unavailable for all TFs (Table 3, Table 4 and Table 5). This poor characterization of the TFs’ dimeric partners results in uncertainty about the biological significance of the interaction and is observed for other factors such as MESP2, FERD3L, NEUROG1, ASCL4, and OLIG3. The color code in Table 3, Table 4 and Table 5 indicates purple for dimeric interactions that have only been analyzed with EMSA, green for interactions only tested with in vivo assays, blue for dimers tested only with in vitro approaches, and yellow for interactions tested with both, in vivo and in vitro assays. 

Table 3, Table 4 and Table 5 and Tables S2–S5 summarize the techniques used to define the bHLH TF homodimeric and heterodimeric interactions. When diverse experimental approaches are used, the interactions can be confirmed unequivocally. Positive or negative interaction results obtained with a particular technique may be influenced by the conditions tested: e.g., whether the proteins were purified, in vitro synthesized, expressed in a specific cell type, co-expressed with other factors, or tested in the presence of DNA or isolated environments. Furthermore, the strength and stability of the interaction tested can also affect the outcome of the experiments [259]. Balancing the available information on the experimental approaches reporting dimeric interactions will help the scientist assess the biological significance of the dimeric interaction of interest. 

Additionally, when experimenting with in vitro translated proteins and recombinant bacteria-synthesized proteins, the protein–protein interactions may not be observed due to the requirement for specific posttranslational modifications or accessory proteins (e.g., LIM-domain proteins). For example, the interaction demonstrated by coIP between ATOH8 and NEUROD1 could not be reproduced using in vitro translated proteins [100]. Similarly, ATOH1 homodimers were confirmed with MS and cell extract EMSAs; however, EMSAs utilizing recombinant proteins did not find the interaction [28,106]. The specific reason for these experimental discrepancies remains to be studied.

In vivo assays preserve the native surrounding in which the interaction takes place. However, these assays also have drawbacks, such as the expression under non-physiological conditions (e.g., heterologous) and the influence of the cell context. Sometimes, an interaction can be observed in one cellular context but not in another. Reasons for these inconsistencies could be a requirement for additional interacting factors or an altered bHLH network composition due to a TF overexpression. TAL1 is an example of a TF capable of activating or repressing transcription in a context-dependent manner through differential interactions with HDACs and HATs [122,123,124] and sequestering E-proteins from other bHLH TFs such as MYOD1 [125].

In the transcriptional reporter assays, the most common approach to define the function of the dimeric bHLH TFs, the main drawback is the presence of a specific endogenous pool of bHLH TFs in the cell. This TF pool may contain TFs able to influence the function of the TF tested, a condition that must be considered by the scientist when analyzing homodimeric TFs.

## 7. Heterodimeric Interactions among bHLH TFs of Classes I, II, V and VI 

In compiling this review, information was gathered about the dimeric interactions of bHLH TFs from individual publications since their discovery at the end of the 1980s. This exhaustive literature search was complemented by a manual search in global protein–protein interaction databases to guarantee a thorough summary of the bHLH dimer diversity. These web databases summarize experimentally corroborated and predicted vertebrate protein–protein interactions, with none of them being devoted to TFs or specifically to bHLH TFs. For this work, IntAct [261], String [262], the Bioplex Interactome [263], and the human interactome database [264] were queried. Only the experimentally confirmed interaction data were included. 

Table 3 (parts A and B) shows heterodimeric interactions among all Class II TFs and the E-proteins TCF4, TCF12, and the two most prominent alternative splicing variants of TCF3: E12 and E47. It was found that 87% of the tissue-specific TFs can interact with either E12 or 47. Furthermore, 30% of the class II TFs can interact with all three E-proteins. It derives from here that E-proteins can replace each other’s functions. However, it is established that individual E-proteins are better partners than others for specific Class II TFs [25,26,27,28,29,30,31,32,33,34,35]. The E-protein–Class II TF interactions are the best characterized in the family and are generally considered to support transcription. However, Table 2 shows that multiple tissue-specific factors can sequester E-proteins and result in adverse transcriptional effects. Furthermore, gaps in Table 3 exemplify interactions that remain to be tested. 

Class I TFs can also heterodimerize among each other, still functioning as transactivators (Table S2). Unfortunately, these heterodimers are poorly characterized, and there are no reports yet comparing the functionality of Class I homodimers with heterodimers. 

Besides heterodimerizing with E-proteins, some bHLH TFs also form heterodimers with other Class II, V, and VI TFs (Table 4 parts A and B, and Table S3). This diversity of dimeric interactions alters the TFs functionality accordingly. For example, MYOD1 functions as a transactivator when heterodimerizing with E47 or E12 [50,66]. However, MYOD1 cannot transactivate as a homodimer [69] or when heterodimerizing with TWIST1 [81], bHLHE41 [154,221], HEY1 [101], and ID proteins [20,183]. ASCL1 homodimers and heterodimers with NEUROG2, HELT, or E12 function as transactivators [107,114,244]. However, experimental evidence exists for heterodimeric interactions between ASCL1 and HAND1 [90] or HES5 [170]. These heterodimers block the activity of ASCL1.

From analyzing Table 4, 91% of the Class II–Class II TF heterodimers are transcriptionally inactive or repressive (Table S4). The only exceptions are the ASCL1/NEUROG2 dimer that transactivates *Dll3* [107] and TAL1/LYL1 [127,245]. These two heterodimers transactivate through cooperation with additional factors [127,245]. The Class II factors TWIST1 [80,82], HAND1 [88,89,90], and TAL1 [125] can sequester other Class II factors. OLIG2 is a Class II protein with a repressor domain that can repress other Class II factors [148,150], homodimerize, or heterodimerize with E-proteins [146,150]. 

In contrast, 100% of Class II TFs interactions with ID proteins and 95% of the interactions with Class VI TFs, negatively affect transcription (Table 4 and Table S3). The Class V family, characterized by the absence of the basic DNA-binding domain, operates by sequestering E-proteins in non-DNA-binding heterodimeric complexes (Table S3). ID proteins can also establish non-functional dimers with Class II and VI TFs (Table S3). The Class VI family groups repressors [22,265], which structure homodimers and heterodimers with TFs from Classes II, V, and VI (Table S3). For instance, the best-characterized family member, HES1, heterodimerizes with multiple partners to block their activity or form dimeric repressors. These partners include E-proteins, MYOD1, PTF1, NHLH2, HEY1, HEY2, HEYL, and IDs (Table S3). Thus, whereas Class II factor interactions with E-proteins can generate transactivators or titrate Class I TFs, Class II TFs’ interactions with Class V, VI, and other Class II factors generally interfere with transcriptional activation. 

The comprehensive 2019 review by Murre [6] summarized the detailed role of bHLH TFs in various pathways and clearly stated that the dynamics of the bHLH gene expression dictates the developmental choice. For many bHLH TFs, though, it did not emphasize the dimeric form involved. Likewise, studies only assessing the regulatory role of a Class II TF as a single entity, with no information about the dimerization partner, are common [251,266,267,268,269,270,271,272,273]. These omissions probably are because the dimeric partner involved is usually a ubiquitous TF such as the E-proteins, which are considered a platform for regulating a broad set of genes [5]. Furthermore, the tissue-specific regulators (Class II TFs) are usually responsible for fine-tuning gene expression, even when heterodimerizing with E-proteins. However, because the dimer composition is very variable (Table 3, Table 4 and Table 5), it is proposed that future publications in the field should state the composition of the dimeric (or multimeric) bHLH TFs involved. As an example, in a study of the role of the bHLH TF SCX in tissue fibrosis, it was concluded that the relevant bHLH dimer is SCX/E47, as SCX by itself did not have a role in the experiments tested [274].

## 8. bHLH TF Homodimers

Homodimerizing tissue-specific bHLH proteins in *Drosophila* were described as transcriptionally inactive [275,276]. These TFs became active upon heterodimerization with E-proteins. MYOD1 could also exist as a homodimer [39,66]. Experiments demonstrating MYOD1 homodimerization include X-ray crystallography [250], CD [231], Y2H [183], and LC-MS/MS [135]. The MYOD1 homodimers were transcriptionally inactive because their duplex DNA binding was compromised [8]. It was later established that MYOD1 and other myogenic bHLH TF homodimers preferred to bind quadruplex DNA instead of duplex DNA [69]. These DNA structures are now known to have a biological role, usually interfering with transcription [277]. Unfortunately, only the myogenic factors have been tested for binding this type of DNA structure [69]. Thus, further research in this area is required to determine whether G-quadruplex binding is a common way of inhibiting transcription by bHLH homodimers.

No systematic reviews about bHLH TF homodimerization exist. Table 5 summarizes the available homodimer information for 5 Class I and 48 vertebrate Class II bHLH TFs. From there, 21 Class II factors have experiments supporting homodimerization through consistent results using in vivo and in vitro approaches (yellow). Table 5 also indicates whether the experimental evidence supports (Y) or does not support (N) homodimers or when the experimental evidence is inconclusive (question mark).

Homodimers of the myogenic factors (MYOD1, MYOG, MYF6) and six other factors (ASCL3, BHLHE22, MSC, OLIG2, BHLHE40, and BHLHE41) are transcriptionally inactive or act as repressors. Within this group, only ASCL3 does not bind duplex DNA as a homodimer. In contrast, five TFs bind DNA to remain transcriptionally inactive or repress gene expression (MSC, BHLHE22, OLIG2, BHLHE40, and BHLHE41). Thus, some Class II homodimers affect gene expression negatively through diverse mechanisms that can be dependent or independent of DNA binding, including the possible sequestration of components of active dimers.

BHLHA15 homodimers can transactivate or repress transcription through direct DNA binding in a context-dependent manner [115,116,254]. HAND1 and HAND2 homodimers cannot bind DNA. However, there is inconclusive evidence about their transcriptional roles [85,91,199].

Six Class II homodimers can function as transactivators through direct DNA binding: NEUROD6, NEUROG2, BHLHA15, ASCL1, NHLH1, and NHLH2. Thus, Class II homodimers can be both transcriptionally inactive and active. On the other hand, homodimerization of 27 (of 48) class II TFs is uncertain, either because it has never been tested or because the experimental evidence is inconclusive. The untested factors include FIGLA, NEUROD1, NEUROD2, ATOH8, ASCL4, ASCL5, TCF23, TCF24, BHLHE23, OLIG1, and OLIG3 (gray in Table 5). Similarly, homodimeric interactions for 16 Class II factors are inconclusive because interactions have only been tested utilizing EMSA (purple) or because independent experiments are insufficient.

Table S5 enlists homodimeric interactions for 4 Class V and 11 Class VI bHLH TFs. All Class V proteins are considered not to homodimerize. However, a splicing variant of ID1, ID1.25, was observed in adult cardiac myocytes and vascular smooth muscle cells [246]. ID1.25 preferentially forms homodimers and probably regulates the sequestering activity of ID1 [246].

Finally, 9 of 11 Class VI factors function as homodimers repressing transcription (Table S5). Homodimerization for HES7 has not been tested, and the evidence for HESL homodimers remains inconclusive. Class VI factors can repress by DNA binding or sequestration of other factors when structuring heterodimers [15,278].

## 9. Conclusions

**Figure 2:**
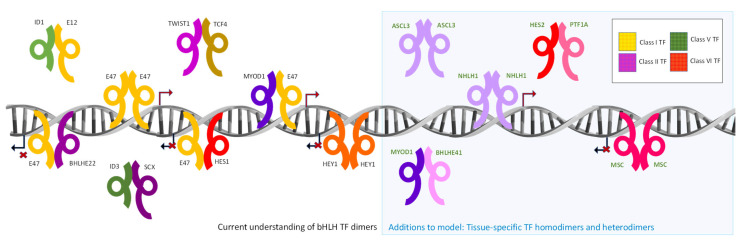
The diversity of dimeric interactions among different bHLH TF classes. Examples of dimeric interactions formed between bHLH TF members of different classes are shown. Examples of the proposed additions to the current model of bHLH TF dimers are highlighted in a blue area. Folded arrows in DNA indicate transactivating or repressing abilities. Interactions independent of DNA binding usually block transcription by factor sequestration.

Although bHLH TFs have been studied for over 30 years, there remain extensive gaps in our knowledge either because some dimeric interactions have never been tested or due to the inherent limitations of the techniques used. Furthermore, due to the bHLH TFs’ ability to interact with multiple partners, dissecting the function of each dimer pair requires carefully designed experiments. I anticipate that the field will be accelerated by increasingly powerful technologies such as cryo-electron microscopy/tomography and genome-wide interactomes in different cell types and conditions. Another layer of complexity is added by the fact that alternate dimerizing partners are usually co-expressed in vivo, establishing a dynamic pool of TFs, whose balance defines the outcome of the assays. This indicates the need for more live-cell approaches to allow the visualization of interaction dynamics and computational approaches using available dimer structures to predict bHLH interactions [279,280] and study the energy of the interaction landscape for bHLH homodimers and heterodimers [281,282]. Keeping this in mind, the available data presented in the tables support two major additions to the current functional bHLH TFs model (Figure 2). First, Class II factors’ interactions with bHLH TFs other than E-proteins usually result in adverse transcriptional effects. Second, homodimers of Class II TFs are common and have both positive and negative transcriptional effects. Positive effects are associated with DNA binding, whereas adverse effects can be independent of or dependent on DNA binding, including binding to G-quadruplex DNA structures. Because the study of bHLH TFs is such an active and productive field of investigation, the years ahead will likely bring ever-increasing insight into sophisticated networks of gene regulation contributing to human development, health, and disease.

## Figures and Tables

**Table 1:** bHLH TFs classification used in this work.

bHLH Class	Characteristic	Homodimerization	Heterodimerization	Examples	Activity	PDB ID ^+^
I	E proteins	Yes	Classes I, II, V, and VI TFs	TCF3, TCF4	A	6OD4
II	Tissue specific	Yes	Classes I, II, V, and VI TFs	NEUROD1, TWIST1	A or R	2QL2
III	LZ domain	*	Classes III and IV	MYC, SRBEF1	A or R	2A93
IV	LZ domain	*	Classes III and IV	MAD, MAX	A or R	1R05
V	No basic domain	No	Classes I, II, V, and VI	ID1, ID4	R	6MGN
VI	Proline in the basic domain	Yes	Classes I, II, V, and VI	HES1, HEY1	R	2MH3
VII	PAS domain	No	Class VII	ARNT, HIF1A	A or R	5SY7

* Few members can homodimerize, including SRBEF1 and MAX [37]. Dimeric interactions for classes in gray are not summarized in this review. A: transactivator; R: transcriptional repressor. ^+^ Example of a Protein Data Bank (PDB) ID [38]. Class II homodimers and heterodimers are summarized in detail in this work. Refs. [6,21,22,37,39,40,41].

**Table 2:** bHLH TFs’ classification and function.

HGNC Gene Symbol and Aliases (a)	Classification			Function	Organism (b)
	[42,44]	[21,22,36]	[47,48]	As homodimers and/or heterodimers	
**Class I**					
TCF3/E47 (E2-5)/ITF1	Group A	Class I	BHLH2/B	E2A: A [52,53,54,55], E2-5 [56], E47 [57]. CR [33,50,58,59]	*Hs, Mm, Rn*
TCF3/E12	Group A	Class I	BHLH2/B	A [33,50,57,60]	*Hs, Mm, Rn, Gg*
TCF4/E2-2A/ITF2	Group A	Class I	BHLH2/B	A [52,53,56,57,60,61]. CR [62,63]	*Hs, Mm*
TCF4/E2-2B	Group A	Class I	BHLH2/B	CR [62,63]	*Hs, Mm*
HEB/TCF12	Group A	Class I	BHLH2/B	A [52,57,60,64]	*Hs, Mm*
**Class II**					
MYOD1/MYOD/MYF3	Group A	Class II	BHLH3/C	A [65,66,67,68]. TI [69]	*Hs, Mm, Rn, Gg*
MYOG/MYF4/Myogenin	Group A	Class II	BHLH3/C	A [49,70]. TI [69]	*Hs, Mm, Rn*
MYF5	Group A	Class II	BHLH3/C	A [71,72]	*Hs, Mm*
MYF6/MRF4/Herculin	Group A	Class II	BHLH3/C	A [49,68,70,73]. TI [69]	*Hs, Mm, Rn, Gg*
MESP1/BHLHC5	Group A	Class II	BHLH3/C		*Mm*
MESP2/BHLHC6	Group A	Class II	BHLH3/C		*Mm*
FIGLA/FIGα/BHLHC8	Group A	Class II	BHLH3/C	A [74,75]	*Hs, Mm*
SCX/Scleraxis	Group A	Class II	BHLH1/A	A [76,77,78]	*Hs, Mm, Rn, M*
TCF15/Paraxis/Meso1	Group A	Class II	BHLH1/A	A [79]	*Hs, Mm*
TWIST1	Group A	Class II	BHLH1/A	R [80,81,82]	*Hs, Mm, Gg*
TWIST2/DERMO1	Group A	Class II	BHLH1/A	R [82,83]	*Hs, Mm*
FERD3L/NTWIST	Group A	Class II	BHLH1/A	R [84]	*Hs, Mm, Dm*
HAND1/EHAND/Thing1	Group A	Class II	BHLH1/A	A [85,86]. R [86,87,88,89,90]	*Mm*
HAND2/DHAND/Thing2	Group A	Class II	BHLH1/A	A [91]. CR [90,92]	*Mm*
PTF1A/P48	Group A	Class II	BHLH1/A	A [93,94]	*Hs, Mm, Rn, M*
NEUROD1/BETA2/NEUROD	Group A	Class II	BHLH1/A	A [29,95,96,97,98,99]	*Hs, Mm, Ma, Rn, Xl*
NEUROD2	Group A	Class II	BHLH1/A	A [97,100]	*Hs, Mm*
NEUROG1/NGN1/NEUROD3/Neurogenin1	Group A	Class II	BHLH1/A	A [97]. R [101,102]	*Hs, Mm, Rn, Xl, Gg* (c)
NEUROD4/ATOH3/MATH3/NeuroM	Group A	Class II	BHLH1/A	A [97,103]	*Mm, Gg, Xl*
NEUROD6/ATOH2/MATH2/NEX1	Group A	Class II	BHLH1/A	A [97,104]. R [105]	*Hs, Mm, Rn*
ATOH1/MATH1	Group A	Class II	BHLH1/A	A [28,97,106]	*Mm*
NEUROG2/ATOH4/MATH4A/Neurogenin2	Group A	Class II	BHLH1/A	A [97,107]. R [31]	*Mm, Gg*
NEUROG3/ATOH5/MATH4B/Neurogenin3	Group A	Class II	BHLH1/A	A [97,108]. CR [109]	*Mm, Hs*
ATOH7/MATH5	Group A	Class II	BHLH1/A	A [110,111]	*Hs, Mm, Gg*
ATOH8/MATH6	Group A	Class II	BHLH1/A	wA, wR [100,112,113,114]	*Hs, Mm*
BHLHA15/MIST1	Group A	Class II	BHLH1/A	A [115]. R [116]	*Hs, Rn, Mm*
ASCL1/MASH1	Group A	Class II	BHLH1/A	A [100,107,117]. R [111]	*Mm, Rn, Gg*
ASCL2/MASH2	Group A	Class II	BHLH1/A	A [117]. CR [118]	*Mm, Rn, Hs*
ASCL3/SGN1	Group A	Class II	BHLH1/A	R [119]	*Hs, Mm*
ASCL4/HASH4	Group A	Class II	BHLH1/A		*Hs*
ASCL5	Group A	Class II	BHLH1/A		*Hs*
TAL1/SCL	Group A	Class II	BHLH1/A	CA, CR [120,121,122,123,124,125]	*Hs, Mm*
TAL2	Group A	Class II	BHLH1/A	Predicted similar to TAL1 [126]	*Hs, Mm*
LYL1	Group A	Class II	BHLH1/A	A [127,128]. R [129]	*Hs, Mm*
NHLH1/HEN1/NSCL	Group A	Class II	BHLH1/A	A, R [130]	*Hs, Mm*
NHLH2/HEN2/NSCL2	Group A	Class II	BHLH1/A	A [131]. R [132]	*Hs, Mm*
MSC/Musculin/ABF-1/MyoR	Group A	Class II	BHLH1/A	R [133,134,135]	*Hs, Mm*
TCF21/Capsulin/POD1	Group A	Class II	BHLH1/A	A, R [136,137,138,139]	*Hs, Mm*
TCF23/OUT	Group A	Class II	BHLH1/A	R [140]	*Mm*
TCF24/OUT2	Group A	Class II	BHLH1/A		*Hs*
BHLHA9/Fingerin/BHLHF42	Group A	Class II	BHLH1/A	R [141]	*Hs, Mm*
BHLHE22/BHLHB5/BETA3	Group A	Class II	BHLH5/E	R [142,143,144]	*Hs, Mm, Ma*
BHLHE23/BHLHB4/BETA4	Group A	Class II	BHLH5/E	R [145]	*Mm*
OLIG1	Group A	Class II	BHLH5/E	A [146]. R [147]	*Hs, Mm*
OLIG2	Group A	Class II	BHLH5/E	A [148]. R [149,150]	*Mm, Gg, Rn*
OLIG3	Group A	Class II	BHLH5/E	R [151]	*Mm*
BHLHE40/SHARP2/STRA13/DEC1	Group A	Class II	BHLH5/E	R [152,153]	*Hs, Mm, Rn*
BHLHE41/SHARP1/DEC2	Group A	Class II	BHLH5/E	R [154,155]	*Hs, Mm, Rn*
Class V					
ID1	Group D	Class V	BHLH2/B	R [20,156]	*Mm, Hs, Rn*
ID2	Group D	Class V	BHLH2/B	R [157]	*Mm, Hs*
ID3	Group D	Class V	BHLH2/B	R [158]	*Mm, Hs*
ID4	Group D	Class V	BHLH2/B	R [159]	*Mm*
Class VI					
HEY1/HRT1/CHF2/HERP2/Hesr1	Group E	Class VI	BHLH2/B	R [160,161]	*Hs, Mm*
HEY2/HRT2/CHF1 gridlock/HERP1	Group E	Class VI	BHLH2/B	R [161,162]	*Hs, Mm, Rn, Gg*
HEYL/HERP3/HRT3	Group E	Class VI	BHLH2/B	R [163,164]	*Hs, Mm*
HES1/HRY/Xhairy1	Group E	Class VI	BHLH2/B	A [165]. R [106,166,167,168]	*Hs, Mm, Rn, M*
HES2	Group E	Class VI	BHLH2/B	R [12]	*Rn, Xl*
HES3	Group E	Class VI	BHLH3/C	R [166]	*Mm, Hs*
HES4/Xhairy2	Group E	Class VI	BHLH2/B	R [169]	*Xl, Hs* (d)
HES5/ESR9	Group E	Class VI	BHLH2/B	R [168,170]	*Rn, Mm, Xl, Gg*
HES6	Group E	Class VI	BHLH3/C	R [171,172]. Inhibits Hes1 [173,174]	*Hs, Mm, Xl*
HES7	Group E	Class VI	BHLH2/B	R [175]	*Hs, Mm*
HELT/MGN/HESL/MEGANE	Group E	Class VI	BHLH2/B	R [176]	*Mm, Hs, Rn*
**Color key:**					
Binds DNA as homodimer				Transactivator and repressor	
Titrates E-proteins				Transactivator (A)	
Titrates E-proteins and binds DNA as homodimer				Repressor (R)/Context dependent repressor (CR) Transcriptionally inactive (TI)	

(a) The HUGO Gene Nomenclature Committee (HGNC) approved gene symbol is followed by common synonyms and aliases, including names for other vertebrates. (b) *Gg* (*Gallus gallus*), *Hs* (*Homo sapiens*), *Mm* (*Mus musculus*), *Rn* (*Rattus norvegicus*), *Xl* (*Xenopus laevis*), *M* (monkey). (c) Sequesters TFs other than E-proteins. (d) No active *Mm* expression.

**Table 3:** Heterodimeric interactions among Class II bHLH TFs and E-proteins. Parts A and B.

Part A.	Heterodimers with E47 or E12	
Class II TFs/Eprot	E47	E12
MYOD1	E2A: MS. Er(4), Ek(2), Ei(4), Ee(2), C(2), cIP(2), FS, qY2H, Sd, GST, Y2H, NI	Ei (5), Er, Ek, Ee, C(3), MIF, cIP(2), qY2H, Y2H, MS, NI, ChIP
MYOG	Er, Ek, Ei, cIP, qY2H [26,69,183]	Ei(3), Er, ChIP MIF, cIP, Y2H, MS [26,64,73,134,135,184,185]
MYF5	Ei, cIP, ChIP, qY2H [26,183,185]	Ei(2), cIP, MIF, ChIP, qY2H, MS [26,72,135,183,185]
MYF6	Er, Ek, Ei, cIP, qY2H [26,69,183]	Ei, MIF, cIP, ChIP, qY2H [26,183,185]
MESP1	Y2H [186]	
MESP2	Y2H [186]	
FIGLA	E2A: Ee(2) [74,75]. Y2H [187]	Ee [74]
SCX	cIP(2), Ee(2), Y2H(2), ChIP, Sd [77,78,188,189]	Er, Ei(2), Y2H(2), MS [76,135,184,190]
TCF15	Ei, Y2H, Sd [186,188,191]	Ei (2), cIP [79,190]
TWIST1	E2A: cIP [192], F, MS [193,194]	GST, cIP, Ei(3), cE, C [80,81,195,196,197]
TWIST2	Sd, GST [157,188,193]	Ei, Y2H [76,184]
FERD3L		Ei, M2H [84]
HAND1	GST, Er, Ei(Dbox)(2), cE, C, cIP(2), F [85,86,87,89,198]	Ei (2)(Dbox), cIP(3), F, cE [87,88,198,199]
HAND2	E2A: Y2H, GST, Ee [30]. Y2H, Ei, cIP, F, M2H [91,157,193]	GST, Y2H, M2H, Ei(2), C [91,197]
PTF1A	MDS, Ei [93,200]	Ee, Ei(3) [93,94,201]
NEUROD1	Ee(2), Ei, Er, Cr, NI [29,95,98,143,202,203,204]	Ei (3), Ee (2), Er, GST [29,95,98,205,206,207]
NEUROD2		cIP, Ei [25]
NEUROG1	Ei [101]	
NEUROD4	Y2H, Er [208]	
NEUROD6	ChIP, Er [104,209]	
ATOH1	TCF3:MS [106,210]	
NEUROG2	cIP, ChIP, Y2H, GST [31,150]	GST, Ei(2), cIP [107,206]
NEUROG3	Ei [108]	Ei [211]
ATOH7	ELISA [212]	
ATOH8	cIP, MS [114,194]	
BHLHA15	Ei, Er, cE, GST, MS [115,116,194]	Ei [213]
ASCL1	TCF3: cIP [35]	Ei(2), Er, Y2H, CD, Ek, cIP [107,184,206,214,215]
ASCL2	cIP, Ei [89]	Ei [117]
ASCL3	Y2H, GST, Ei, C [119]	Y2H, GST [119]
ASCL4		
ASCL5		
TAL1	E2A: Ei, GST, Y2H, cIP, MS. Ee, Ei(2),C, cIP, ChIP, Cr, Y2H.	Er, GST(2), SEC/MALLS [177,216]
TAL2	Ei [217]	Y2H [184]
LYL1	E2A: cIP(2), Ee, GST [27,129]. cIP, ChIP [127]	Ei [26]
NHLH1	GST, M2H, Ee [130,218]	Ei(2), GST [107,218]
NHLH2	*	
MSC	Y2H, Ei [133]	Ei(3), Y2H, MS, GST, cIP [133,134,135]
TCF21		Ei, Y2H(2), M2H, IF [138,219,220]
TCF23		cIP, cE [140]
TCF24		
BHLHA9	E2A: Y2H [141]	
BHLHE22	cIP, cE [143]	cIP, cE [143]
BHLHE23	(b)	
OLIG1	TCF3: cIP (2) [35,146]	cIP [146]
OLIG2	Y2H, GST, Ei, cIP(2) [35,146,150]	cIP [146]
OLIG3		
BHLHE40	Sd, Y2H, cIP [188]	w: GST [152]
BHLHE41	cIP, cE, GST [154,221]	
**Part B. Heterodimers with TCF4 or TCF12**		
Class II TFs/Eprot	TCF4	TCF12
MYOD1	Ee, Ei, cIP, MS, Fw [26,66,125,135]	Ei, MS [64,135]
MYOG	Ei(2), cIP [26,64]	Ei [64]
MYF5	Ei, cIP, ChIP [26,185]	
MYF6	Ei, cIP, ChIP [26,185]	
MESP1		
MESP2		
FIGLA	Ee [75]	Ee [75]
SCX	Y2H [189]	
TCF15		
TWIST1	MS, GST [222,223]	
TWIST2		
FERD3L		
HAND1	MS [222]	Ei, Y2H, cIP (Dbox) [87,88]
HAND2	Y2H(2), GST, Ee, Ei, M2H [30,91,157]	Y2H, GST, Ee(nDB) [30]
PTF1A		Ei (2) [93,94]
NEUROD1	Ee [29]	Ee [29]
NEUROD2	Ei(2), NI, cIP [25,224]	cIP, Ei [25]
NEUROG1		
NEUROD4		
NEUROD6		
ATOH1	cIP, Y2H, Ee [28]	MS [210]
NEUROG2	(a)	(a)
NEUROG3		
ATOH7		
ATOH8		
BHLHA15	MS [222]	
ASCL1	Ei, NI, cIP, M2H [35,157,224]	cIP [35]
ASCL2	Ei [89]	Ei [89]
ASCL3	Y2H, GST [119]	Y2H [119]
ASCL4		M2H [36]
ASCL5		
TAL1	Ei, C, GST, Fw, qY2H [125,183,225]	Ei, C, MS [124,225]
TAL2	qY2H [183]	
LYL1	qY2H [183]	
NHLH1		
NHLH2		
MSC	Y2H [133]	Y2H [133]
TCF21	Y2H(2), GST, cIP [219,220]	Y2H(2) [219,220]
TCF23		M2H [36]
TCF24	MS (2) [222,226]	
BHLHA9	Y2H [141]	Y2H [141]
BHLHE22		
BHLHE23		
OLIG1	cIP [35]	cIP [35]
OLIG2	cIP [35]	
OLIG3		w:M2H [36]
BHLHE40		
BHLHE41		
		
**Color key:**		
	Both in vitro and in vivo	(Independent experiments)
	Only in vitro assays	
	Only EMSA	
	Only in vivo assays	

* Due to numerous references for MYOD and TAL1 interactions, references are indicated here: MYOD-E47: E2A: MS [135]. Er(4), Ek(2), Ei(4), Ee(2), C(2), cIP(2), FS, qY2H, Sd, GST, Y2H, NI [7,26,54,66,67,68,69,183,188,227,228,229,230,231,232]. MYOD-E12: Ei (5), Er, Ek, Ee, C(3), MIF, cIP(2), qY2H, Y2H, MS, NI, ChIP [7,26,67,135,183,184,185,195,227,228,230,232,233]. TAL1-E47: E2A: Ei, GST, Y2H, cIP, MS [124,216,234]; Ee, Ei(2),C, cIP, ChIP, Cr, Y2H [120,127,181,225,234,235]. Numbers in parenthesis indicate independent experiments. Techniques (defined in Appendix A): Er = EMSA with recombinant protein. Ei = EMSA with in vitro translated protein. Ee = EMSA cell/nuclear extracts and super-shift or transfected cells. Ek = EMSA dissociation kinetics. cE = competitive EMSA. cIP: co-immunoprecipitation. GST = GST pulldown and related techniques. C = CASTing. Sd = sandwich assay. IF = colocalization by immunofluorescence. Cr = crystallography. Fw = far Western blot. CD = circular dichroism. F = FRET. FP = DNase I footprinting. w: weak interaction. nDB: no DNA binding. (a) No direct interaction was tested, but it is assumed from transactivation assays with a reporter gene [107]. (b) No direct proof of interaction suggested specific squelching mechanisms through reporter gene repression assay [145]. * The bHLH motif (66 amino acids) only has a one aa difference with HEN1, suggesting that it is also likely to heterodimerize with E2A [218].

**Table 4:** Heterodimeric interactions of Class II bHLH TFs with Classes II, V and VI TFs. Parts A and B.

Part A. Class II-Class II Interactions														
	MYOD1	MYOG	MYF5	MYF6	TWIST1	HAND1	HAND2	NEUROD1	NEUROG2	NEUROG3	ASCL1	TAL1	OLIG1	BHLHE40
MYOD1														
MYOG														
MYF5														
MYF6					GST (71)									
MESP1														
MESP2														
FIGLA														
SCX														
TCF15														
TWIST1	cIP, GST [81]	GST [81]	GST [81]	GST [81]	x									
TWIST2					MS [236]									
FERD3L														
HAND1	M2H [90]	x			cIP [193]									
HAND2					cIP, F [193]	*	x	x						
PTF1A														
NEUROD1														
NEUROD2														
NEUROG1														
NEUROD4														
NEUROD6														
ATOH1														
NEUROG2														
NEUROG3														
ATOH7														
ATOH8								cIP [100]	x	cIP [100]				
BHLHA15	Ei (nDB), GST [116]	x												
ASCL1						GST [90]	x		**	x				
ASCL2						cIP [88]	x							
ASCL3	GST [119]	x												
ASCL4														
ASCL5														
TAL1														
TAL2														
LYL1												****		
NHLH1														
NHLH2														
MSC														
TCF21														
TCF23														
TCF24														
BHLHA9					w: Y2H [141]		Y2H [141]	x						
BHLHE22														
BHLHE23														
OLIG1														x
OLIG2									***	x			cIP, M2H [148]	
OLIG3														
BHLHE40	cIP [155]	x									GST (nDB) [152]	x		
BHLHE41	cIP(2), cE, GST(2)	x												GST [237]
**Part B. Class II-Class V or VI interactions**														
	ID1	ID2		ID3		ID4	HEY1	HEY2	HEYL	HES1	HES2	HES4	HES5	HELT
MYOD1	*****	qY2H, cIP, M2H [183]		cIP, Y2H, cEMSA [158]			cIP, cE [160]	x		w: SSPC [164]	x			
MYOG														
MYF5	qY2H, M2H [183]	qY2H, M2H [183]		M2H [183]		x								
MYF6	cIP [183]	cIP [183]		cIP [183]		x								
MESP1														
MESP2														
FIGLA														
SCX				cIP [238]		x								
TCF15														
TWIST1	cIP [192]	x		cIP [192]		x						cIP [239]	x	
TWIST2														
FERD3L														
HAND1							GST [90]	GST [90]	GST [90]	x				
HAND2							GST [90]	GST [90]	GST [90]	x				
PTF1A				MDS [200]		x	cIP [240]	cIP [240]	x	cIP, Y2H, GST [240]	x			
NEUROD1							w: cIP [241]	x			cIP [242]	x	cIP [241]	x
NEUROD2														
NEUROG1														
NEUROD4							w: cIP [241]	x			cIP [242]	x	cIP [241]	x
NEUROD6														
ATOH1														
NEUROG2													cIP [241]	x
NEUROG3														
ATOH7														
ATOH8														
BHLHA15														
ASCL1	x												cIP(2) [170,243]	+++
ASCL2														
ASCL3														
ASCL4														
ASCL5														
TAL1														
TAL2														
LYL1														
NHLH1														
NHLH2										GST, cIP [132]	x			
MSC														
TCF21														
TCF23														
TCF24														
BHLHA9														
BHLHE22														
BHLHE23														
OLIG1		cIP, b2H, IF [146]		x		+	x							
OLIG2		cIP, b2H, IF [146]		x		++	x							
OLIG3														
BHLHE40	x						x							
BHLHE41														

Color keys and abbreviations are as in Table 3. The pale-yellow area does not show interactions to avoid repeated data from the white area. Interactions with numerous references are detailed here: BHLHE41-MYOD1: cIP(2), cE, GST(2) [154,155,221]. * HAND2-HAND1: Y2H, GST, F, M2H [90,193]. ** ASCL1-NEUROG2: Y2H, cIP, GST, Ei(2), Ee [107,206,244]; for [206], no DNA binding was observed with EMSA with in vitro translated protein. *** OLIG2-NEUROG2: cIP, M2H, Y2H, GST [148,150]. **** LYL1-TAL1: MS, cIP, ChIP, ChIP-seq [124,127,245]. ***** MYOD1-ID1: cIP(3), Y2H, GST, M2H(2), qY2H [157,176,183,244,246]. + OLIG1-ID4: BMFCS [247], cIP, b2H, IF [146]. ++ OLIG2-ID4: cIP, b2H, IF [146]. +++ ASCL1-HELT: Y2H, cIP, IF [244].

**Table 5:** Homodimeric interactions of Class I and II bHLH TFs.

Factor	Homodimer	EMSA	2H	GST	cIP	MS	Biophysical	Other	Function (a)
**Class I**								Biochemical	
TCF3/E47	Y	Y: Ei [7,8], Er [227], Ee (2) [30,54]				Y: [194]	Y: Cr [248], FS, CD [231]	Y: C, Ek [227]; MIF [8]	A [52,53,54,55,56,57]
TCF3/E12	Y	*	Y: Y2H [184]			Y: [135]	Y: CD [177]	Y: C, Ek [227] (b)	A [52,57,60]
TCF4	Y	Y: Er [249], Ei [224]				Y: [222]	Y: Cr, (f) [249]		A [52,56,57,60,61]
TCF12	Y	Y: Ei [64]							A [52,57,60]
**Class II**									
MYOD1 (e)	Y	**	w: qY2H [183]			Y: [135]	Y: Cr [250], CD, FS [231]	Y: MIF [70]	TI [69]
MYOG (e)	Y	Y: Er (2) [72,185], wq: Er [69]	w: qY2H [183]					Y: MIF [70]	TI [69]
MYF5 (e)	Y	Y: Er [72]	w: qY2H [183]					Y: MIF [70]	
MYF6 (e)	Y	w: Er [70], q: Er [69]	N: qY2H [183]						TI [69]
MESP1	?		N: Y2H [186]						
MESP2	?		N: Y2H [186]						
FIGLA	?								
SCX	?	N: Ei, Er [76]; Y: Ei [251]							A [251]
TCF15	?	N: Ei (3) [79,190,191]	N: Y2H [186]						
TWIST1	Y	Y: Ei (2) [196,197]		Y: [81]	Y: [236]	Y: [236]		Y: FRET [193], (g) [196]	
TWIST2	?	N: Ei [184]							
FERD3L	?	N: Ei [84]							
HAND1	Y	N: Er [86], Ei [87]	Y: M2H, Y2H [90]	Y: [90]	Y: [89,90]			Y: FRET [252], C(nDB) [90]	A? [85,199]
HAND2	Y	N: Ei [91], Ee [30]	Y: Y2H w: M2H [91]	Y: [90,91]	Y: [92]			N: C [91]	TI? [91]
PTF1A	?	N: Ei [94,201], Ee[93].							
NEUROD1	?								
NEUROD2	?								
NEUROG1	?						Y fuzzy E-box: CD [253]		
NEUROD4	?	N: Ei [208]							A? [103]
NEUROD6	Y?	Y: Er [105], Ee [104]							A [105]
ATOH1	Y	N: Er [106]. Y: Ee [28]				Y: [210]			
NEUROG2	Y	N: Ei [206]	N: Y2H [150]		Y: [31]			Y: ChIP [31](c)	A [31] (i)
NEUROG3	N?	N: Ei [108]					N: CD (nDB) [253]		
ATOH7	?	Y: Er [111]						N: ELISA (h) [212]	A [111]
ATOH8	?								
BHLHA15	Y	Y: Ei [115,116,213]; Ee [254]		Y: [116]	Y: [115,254]			Y: C [115], BMFCS [254]	A [115,254]. R [116]
ASCL1	Y	Y: Ei [107]	Y: Y2H [244]		Y: [244]		Y: CD [255]	Y: Ek [214]	A [107]. R [111]
ASCL2	?	N: Ei [89]							
ASCL3	Y	N: Ei [119]	Y: Y2H [119]	Y: [119]				N: C [119]	R [119]
ASCL4	?								
ASCL5	?								
TAL1	Y	N: Ei [216], Er [177]	N: qY2H [183]	N: [125,127]		Y: [124]	Y: CD [177]		
TAL2	?	N: [217]	N: qY2H [183]						
LYL1	Y		N: qY2H [183]	Y: [127]	Y: [127]				
NHLH1	Y	Y: Ei, Ee [130,218]	Y: M2H [130]	Y: [218]				Y: C [218]	A [130]
NHLH2	Y	Y: Ee [131]		Y: [131]					A [131]
MSC	Y	Y: Ei [133,134]							R [133,134]
TCF21	N?	N: Ei [219]	N: Y2H [137,220]						
TCF23	?								
TCF24	?								
BHLHA9	?		Y: Y2H [141]						
BHLHE22	Y	N: [143]			Y: [142]			Y: ChIP [142](d)	R [142,144]
BHLHE23 (c)	?								R [145]
OLIG1	Y	Y: Er [147]							R [147]
OLIG2	Y	Y: Ei [150]	Y: Y2H [150], M2H [148]	Y: [150]	Y: [148]		Y: FCCS [256]		R [149,150,257]
OLIG3	?								
BHLHE40	Y	Y: Ei [153,155,237]			Y: [153]				R [153,155]
BHLHE41	Y	Y: Ei [155,221]		Y: [155]					R [155,221]
**Color key:**	**Experiment**	**DNA binding**							**Function**
	only EMSA	No DNA binding	(Independent experiments)						Transactivator (A)
	only in vitro assays	DNA binding							Repressor (R)
	only in vivo assays	Opposite DNA binding results							A and R
	In vitro and in vivo								(j)
	Untested								

* N: Ei [7] (N), [230] (N), [184] (Y); Er [227] (N); w: Er [67]. ** w: Ei [230], Er (4) [65,72,227,231]. q: Er [69]. N: Ei [7]. (a) Consider the possibility of confounding results due to heterodimerization with endogenous proteins in reporter transfection assays. (b) Chromatographic properties on gel filtration suggest a stable homodimer with no DNA binding by EMSA [227]. (c) There was 95% bHLH sequence identity with bHLHB5 [145]. (d) For BHLHE22 and NGN2, DNA binding was not observed with EMSA but was demonstrated with ChIP. The requirement for additional factors for DNA binding cannot be eliminated. All myogenic factors had weak interactions with duplex DNA but bound quadruplex DNA well. (f) Fluorescence polarization and isothermal titration calorimetry. (g) Non-reducing SDS-PAGE of co-transfected protein extracts. (h) Protein–protein and DNA–protein interaction ELISAs. (i) Low phosphorylation. (j) The factor was tested as a homodimeric transactivator with hard-to-dissect reporter assays due to possible dimerization with endogenous proteins. Techniques: Er = EMSA with recombinant protein. Ei = EMSA with in vitro translated protein. Ee = EMSA cell/nuclear extracts and super-shift or transfected cells. Ek = EMSA dissociation kinetics. cE = competitive EMSA. cIP: coimmunoprecipitation. GST = GST pulldown and related techniques. C = CASTing. Sd= sandwich assay. IF = colocalization by immunofluorescence. Cr = crystallography. Fw = far Western blot. CD = circular dichroism. F = FRET. FP = DNase I footprinting. MS = mass spectrometry. BMFCS = bi-molecular fluorescence complementation system. FCCS = fluorescence cross-correlation spectroscopy. w: weak interaction. nDB: no DNA binding. q: quadruplex DNA.

## Supplementary Materials

The following are available online at https://www.mdpi.com/article/10.3390/ijms222312855/s1.

## Appendix A. Methods for Detecting Protein–Protein Interactions of bHLH TFs

### Appendix A.1. In Vitro Interactions

Electrophoretic Mobility Shift Assay (EMSA, biochemical): Detects protein–nucleic acid interactions through slower electrophoretic mobility of the complex compared to the free nucleic acid [227,230].

The protein tested to interact with DNA can be derived from the following sources:

- Cell/nuclear extracts. Detection of a specific protein in the mixture can be achieved by incubation with antibodies that produce a super-shift or slower electrophoretic mobility in comparison to the protein–nucleic acid complex by itself.
- Recombinant purified proteins
- In vitro transcribed/translated proteins.

The DNA templates tested include the following:

- Single short DNA probes containing one or multiple protein-binding sequences (e.g., E-boxes, N-boxes, or ESE-boxes)
- Multiple random DNA-binding sequences (CASTing: Cyclic Amplification and Selection of Targets) [227,258].

**Competitive EMSA** (cEMSA, biochemical): Detects the disappearance of a shifted band when a protein–nucleic acid interaction is lost by incubation with another protein that can interact with a protein in the DNA–protein complex [143,158].

**GST-Pulldown** (biochemical): A protein fused to GST (bait) is immobilized to capture proteins that interact [283]. Interacting proteins can be recombinant, in vitro translated, or derived from cell lysates. Bait proteins can be immobilized utilizing alternative fused proteins.

**Mass Spectrometry** (MS, biophysical): Analytical chemistry method that measures the mass-to-charge ratio of molecules present in a sample. Proteins are identified from the mass spectra using computational methods [284].

**X-ray Crystallography** (biophysical): Determination of atomic-resolution structures by analyzing the diffraction of X-rays by crystals of a purified protein or complex [285].

**Circular Dichroism** (CD, biophysical): Spectroscopic method that characterizes protein structures by measuring differences in absorption of left and right circularly polarized light [286].

**Footprinting** (biochemical): Detects sequence-specific DNA binding when a purified protein or protein complex protects DNA from degradation by DNase I [287].

**Methylation Interference Footprinting** (MIF, biochemical): Identifies the exact DNA sequence for binding when a protein cannot bind its recognition sequence when DNA is methylated. Chemical cleavage distinguishes unprotected regions from protected sequences [73].

**Enzyme-Linked Immunosorbent Assay** (ELISA, biochemical): DNA–protein and protein–protein interactions can be detected by immobilizing DNA or a bait protein in microwell plates. Binding partners are detected from cell lysates through antibody interactions and enzymatic reactions [212,286].

**Far Western Blot** (biochemical): Proteins blotted to a membrane from an SDS-PAGE are probed for direct interaction with another protein that can be labeled or detected indirectly with specific antibodies [288].

**Sandwich Screening Procedure** (biochemical): Relies on protein–protein interactions to generate a specific DNA-binding activity. Recombinant proteins are immobilized in nitrocellulose and incubated with a “bait” protein. Incubation with labeled E-box probes confirmed the interaction between bHLH heterodimers and DNA [188].

**Co-immunoprecipitation** (co-IP, biochemical): Identifies protein–protein interactions when a bait-specific antibody is used to co-precipitate binding partners [289]. It is also considered an ex-vivo approach as the interaction occurs in vivo, whereas its detection occurs in vitro.

### Appendix A.2. In Vivo Interactions

**Yeast Two-Hybrid** (Y2H, genetic): A reporter gene is activated when a bait protein fused to the DNA-binding domain of the transcription factor Gal4 is interacting with a prey protein fused to the Gal4 activation domain [290].

**Bacterial Two-Hybrid** (B2H, genetic): Adapted Y2H in bacteria. Avoids requirement for nuclear compartmentalization of proteins tested [291].

**Mammalian Two-Hybrid** (M2H, genetic): Adapted from Y2H in mammalian cells. Studies interactions in native context [291].

**Fluorescence Resonance Energy** Transfer (FRET, biochemical): When two proteins are interacting, energy is transferred from a bait protein fused to a donor fluorophore, upon excitation, to a prey protein fused to an acceptor fluorophore [252,286].

**Site-Specific Photocrosslinking** (SSPC, biochemical): proteins of interest incorporate a photocrosslinkable amino acid. In vivo interacting proteins are crosslinked with UV-light, and interactions are detected by IP [164].

**Nuclear Importing/Redirection Assays** (NI, biochemical): A cytoplasmic protein (e.g., due to deletion of the nuclear localization signal) is imported to the nucleus upon interaction with protein imported to the nucleus [232].

**Chromatin Immunoprecipitation** (ChIP, biochemical): Proteins are crosslinked to DNA and precipitated with an antibody. DNA sequence bound can be detected with low- and high-throughput techniques.

**Bimolecular Fluorescence Complementation** (biFC, biochemical): Proteins fused to two split fluorophore fragments can fluoresce upon proximity or interaction [292].
