# Supplementary material for: Homodimeric and Heterodimeric Interactions among Vertebrate Basic Helix–Loop–Helix Transcription Factors
Source: Int J Mol Sci. 2021 Nov 28;22(23):12855. doi: 10.3390/ijms222312855 (PMC8657788; doi:10.3390/ijms222312855)
Supplement: Supplementary file 1 [file ijms-22-12855-s001.zip › Supplementary References Tables S1 S2 S3 and S5 Torres-Machorro.pdf]

Supplementary references for Tables S1, S2, S3 and S5.

1. Zhuang, Y., Cheng, P. and Weintraub, H. (1996) B-lymphocyte development is regulated by the combined dosage of three basic helix-loop-helix genes, E2A, E2-2, and HEB. *Mol Cell Biol*, **16**, 2898-2905.
2. Bain, G., Engel, I., Robanus Maandag, E.C., te Riele, H.P., Voland, J.R., Sharp, L.L., Chun, J., Huey, B., Pinkel, D. and Murre, C. (1997) E2A deficiency leads to abnormalities in alphabeta T-cell development and to rapid development of T-cell lymphomas. *Mol Cell Biol*, **17**, 4782-4791.
3. Beck, K., Peak, M.M., Ota, T., Nemazee, D. and Murre, C. (2009) Distinct roles for E12 and E47 in B cell specification and the sequential rearrangement of immunoglobulin light chain loci. *J Exp Med*, **206**, 2271-2284.
4. Belle, I. and Zhuang, Y. (2014) E proteins in lymphocyte development and lymphoid diseases. *Curr Top Dev Biol*, **110**, 153-187.
5. Perez-Moreno, M.A., Locascio, A., Rodrigo, I., Dhondt, G., Portillo, F., Nieto, M.A. and Cano, A. (2001) A new role for E12/E47 in the repression of E-cadherin expression and epithelial-mesenchymal transitions. *J Biol Chem*, **276**, 27424-27431.
6. Pfurr, S., Chu, Y.H., Bohrer, C., Greulich, F., Beattie, R., Mammadzada, K., Hils, M., Arnold, S.J., Taylor, V., Schachtrup, K. *et al.* (2017) The E2A splice variant E47 regulates the differentiation of projection neurons via p57(KIP2) during cortical development. *Development*, **144**, 3917-3931.
7. Flora, A., Garcia, J.J., Thaller, C. and Zoghbi, H.Y. (2007) The E-protein Tcf4 interacts with Math1 to regulate differentiation of a specific subset of neuronal progenitors. *Proc Natl Acad Sci U S A*, **104**, 15382-15387.
8. Bergqvist, I., Eriksson, M., Saarikettu, J., Eriksson, B., Corneliussen, B., Grundstrom, T. and Holmberg, D. (2000) The basic helix-loop-helix transcription factor E2-2 is involved in T lymphocyte development. *Eur J Immunol*, **30**, 2857-2863.
9. Bouderlique, T., Pena-Perez, L., Kharazi, S., Hils, M., Li, X., Krstic, A., De Paepe, A., Schachtrup, C., Gustafsson, C., Holmberg, D. *et al.* (2019) The Concerted Action of E2-2 and HEB Is Critical for Early Lymphoid Specification. *Front Immunol*, **10**, 455.
10. Cisse, B., Caton, M.L., Lehner, M., Maeda, T., Scheu, S., Locksley, R., Holmberg, D., Zweier, C., den Hollander, N.S., Kant, S.G. *et al.* (2008) Transcription factor E2-2 is an essential and specific regulator of plasmacytoid dendritic cell development. *Cell*, **135**, 37-48.
11. Nagasawa, M., Schmidlin, H., Hazekamp, M.G., Schotte, R. and Blom, B. (2008) Development of human plasmacytoid dendritic cells depends on the combined action of the basic helix-loop-helix factor E2-2 and the Ets factor Spi-B. *Eur J Immunol*, **38**, 2389-2400.
12. Muir, T., Sadler-Riggleman, I., Stevens, J.D. and Skinner, M.K. (2006) Role of the basic helix-loop-helix protein ITF2 in the hormonal regulation of Sertoli cell differentiation. *Mol Reprod Dev*, **73**, 491-500.
13. Skerjanc, I.S., Truong, J., Filion, P. and McBurney, M.W. (1996) A splice variant of the ITF-2 transcript encodes a transcription factor that inhibits MyoD activity. *J Biol Chem*, **271**, 3555-3561.
14. Furumura, M., Potterf, S.B., Toyofuku, K., Matsunaga, J., Muller, J. and Hearing, V.J. (2001) Involvement of ITF2 in the transcriptional regulation of melanogenic genes. *J Biol Chem*, **276**, 28147-28154.
15. D'Cruz, L.M., Knell, J., Fujimoto, J.K. and Goldrath, A.W. (2010) An essential role for the transcription factor HEB in thymocyte survival, Tcra rearrangement and the development of natural killer T cells. *Nat Immunol*, **11**, 240-249.
16. Conway, K., Pin, C., Kiernan, J.A. and Merrifield, P. (2004) The E protein HEB is preferentially expressed in developing muscle. *Differentiation*, **72**, 327-340.
17. Parker, M.H., Perry, R.L., Fauteux, M.C., Berkes, C.A. and Rudnicki, M.A. (2006) MyoD synergizes with the E-protein HEB beta to induce myogenic differentiation. *Mol Cell Biol*, **26**, 5771-5783.
18. Uittenbogaard, M., Martinka, D.L. and Chiaramello, A. (2003) The basic helix-loop-helix differentiation factor Nex1/MATH-2 functions as a key activator of the GAP-43 gene. *J Neurochem*, **84**, 678-688.
19. Weintraub, H., Tapscott, S.J., Davis, R.L., Thayer, M.J., Adam, M.A., Lassar, A.B. and Miller, A.D. (1989) Activation of muscle-specific genes in pigment, nerve, fat, liver, and fibroblast cell lines by forced expression of MyoD. *Proc Natl Acad Sci U S A*, **86**, 5434-5438.
20. Weintraub, H., Davis, R., Tapscott, S., Thayer, M., Krause, M., Benezra, R., Blackwell, T.K., Turner, D., Rupp, R., Hollenberg, S. *et al.* (1991) The myoD gene family: nodal point during specification of the muscle cell lineage. *Science*, **251**, 761-766.

21. Choi, J., Costa, M.L., Mermelstein, C.S., Chagas, C., Holtzer, S. and Holtzer, H. (1990) MyoD converts primary dermal fibroblasts, chondroblasts, smooth muscle, and retinal pigmented epithelial cells into striated mononucleated myoblasts and multinucleated myotubes. *Proc Natl Acad Sci U S A*, **87**, 7988-7992.
22. Hernandez-Hernandez, J.M., Garcia-Gonzalez, E.G., Brun, C.E. and Rudnicki, M.A. (2017) The myogenic regulatory factors, determinants of muscle development, cell identity and regeneration. *Semin Cell Dev Biol*, **72**, 10-18.
23. Braun, T., Bober, E., Buschhausen-Denker, G., Kohtz, S., Grzeschik, K.H. and Arnold, H.H. (1989) Differential expression of myogenic determination genes in muscle cells: possible autoactivation by the Myf gene products. *EMBO J*, **8**, 3617-3625.
24. Braun, T., Buschhausen-Denker, G., Bober, E., Tannich, E. and Arnold, H.H. (1989) A novel human muscle factor related to but distinct from MyoD1 induces myogenic conversion in 10T1/2 fibroblasts. *EMBO J*, **8**, 701-709.
25. Braun, T., Winter, B., Bober, E. and Arnold, H.H. (1990) Transcriptional activation domain of the muscle-specific gene-regulatory protein myf5. *Nature*, **346**, 663-665.
26. Braun, T., Bober, E., Winter, B., Rosenthal, N. and Arnold, H.H. (1990) Myf-6, a new member of the human gene family of myogenic determination factors: evidence for a gene cluster on chromosome 12. *EMBO J*, **9**, 821-831.
27. Kitajima, S., Takagi, A., Inoue, T. and Saga, Y. (2000) MesP1 and MesP2 are essential for the development of cardiac mesoderm. *Development*, **127**, 3215-3226.
28. Takahashi, Y., Hiraoka, S., Kitajima, S., Inoue, T., Kanno, J. and Saga, Y. (2005) Differential contributions of Mesp1 and Mesp2 to the epithelialization and rostro-caudal patterning of somites. *Development*, **132**, 787-796.
29. Liang, L., Soyal, S.M. and Dean, J. (1997) FIGalpha, a germ cell specific transcription factor involved in the coordinate expression of the zona pellucida genes. *Development*, **124**, 4939-4947.
30. Soyal, S.M., Amleh, A. and Dean, J. (2000) FIGalpha, a germ cell-specific transcription factor required for ovarian follicle formation. *Development*, **127**, 4645-4654.
31. Cserjesi, P., Brown, D., Ligon, K.L., Lyons, G.E., Copeland, N.G., Gilbert, D.J., Jenkins, N.A. and Olson, E.N. (1995) Scleraxis: a basic helix-loop-helix protein that prefigures skeletal formation during mouse embryogenesis. *Development*, **121**, 1099-1110.
32. Brown, D., Wagner, D., Li, X., Richardson, J.A. and Olson, E.N. (1999) Dual role of the basic helix-loop-helix transcription factor scleraxis in mesoderm formation and chondrogenesis during mouse embryogenesis. *Development*, **126**, 4317-4329.
33. Blanar, M.A., Crossley, P.H., Peters, K.G., Steingrimsson, E., Copeland, N.G., Jenkins, N.A., Martin, G.R. and Rutter, W.J. (1995) Meso1, a basic-helix-loop-helix protein involved in mammalian presomitic mesoderm development. *Proc Natl Acad Sci U S A*, **92**, 5870-5874.
34. Quertermous, E.E., Hidai, H., Blanar, M.A. and Quertermous, T. (1994) Cloning and characterization of a basic helix-loop-helix protein expressed in early mesoderm and the developing somites. *Proc Natl Acad Sci U S A*, **91**, 7066-7070.
35. Burgess, R., Cserjesi, P., Ligon, K.L. and Olson, E.N. (1995) Paraxis: a basic helix-loop-helix protein expressed in paraxial mesoderm and developing somites. *Dev Biol*, **168**, 296-306.
36. Stoetzel, C., Weber, B., Bourgeois, P., Bolcato-Bellemin, A.L. and Perrin-Schmitt, F. (1995) Dorso-ventral and rostro-caudal sequential expression of M-twist in the postimplantation murine embryo. *Mech Dev*, **51**, 251-263.
37. Spicer, D.B., Rhee, J., Cheung, W.L. and Lassar, A.B. (1996) Inhibition of myogenic bHLH and MEF2 transcription factors by the bHLH protein Twist. *Science*, **272**, 1476-1480.
38. Hebrok, M., Fuchtbauer, A. and Fuchtbauer, E.M. (1997) Repression of muscle-specific gene activation by the murine Twist protein. *Exp Cell Res*, **232**, 295-303.
39. Chen, Z.F. and Behringer, R.R. (1995) twist is required in head mesenchyme for cranial neural tube morphogenesis. *Genes Dev*, **9**, 686-699.
40. Hopwood, N.D., Pluck, A. and Gurdon, J.B. (1989) A Xenopus mRNA related to Drosophila twist is expressed in response to induction in the mesoderm and the neural crest. *Cell*, **59**, 893-903.
41. Cakouros, D., Isenmann, S., Hemming, S.E., Menicanin, D., Camp, E., Zannetinno, A.C. and Gronthos, S. (2015) Novel basic helix-loop-helix transcription factor hes4 antagonizes the function of twist-1 to regulate lineage commitment of bone marrow stromal/stem cells. *Stem Cells Dev*, **24**, 1297-1308.
42. Firulli, B.A., Krawchuk, D., Centonze, V.E., Vargesson, N., Virshup, D.M., Conway, S.J., Cserjesi, P., Laufer, E. and Firulli, A.B. (2005) Altered Twist1 and Hand2 dimerization is associated with Saethre-Chotzen syndrome and limb abnormalities. *Nat Genet*, **37**, 373-381.

43. Firulli, B.A., Milliar, H., Toolan, K.P., Harkin, J., Fuchs, R.K., Robling, A.G. and Firulli, A.B. (2017) Defective Hand1 phosphoregulation uncovers essential roles for Hand1 in limb morphogenesis. *Development*, **144**, 2480-2489.
44. Li, L., Cserjesi, P. and Olson, E.N. (1995) Dermo-1: a novel twist-related bHLH protein expressed in the developing dermis. *Dev Biol*, **172**, 280-292.
45. Verzi, M.P., Anderson, J.P., Dodou, E., Kelly, K.K., Greene, S.B., North, B.J., Cripps, R.M. and Black, B.L. (2002) N-twist, an evolutionarily conserved bHLH protein expressed in the developing CNS, functions as a transcriptional inhibitor. *Dev Biol*, **249**, 174-190.
46. Srivastava, D., Cserjesi, P. and Olson, E.N. (1995) A subclass of bHLH proteins required for cardiac morphogenesis. *Science*, **270**, 1995-1999.
47. Knofler, M., Meinhardt, G., Bauer, S., Loregger, T., Vasicek, R., Bloor, D.J., Kimber, S.J. and Husslein, P. (2002) Human Hand1 basic helix-loop-helix (bHLH) protein: extra-embryonic expression pattern, interaction partners and identification of its transcriptional repressor domains. *Biochem J*, **361**, 641-651.
48. Scott, I.C., Anson-Cartwright, L., Riley, P., Reda, D. and Cross, J.C. (2000) The HAND1 basic helix-loop-helix transcription factor regulates trophoblast differentiation via multiple mechanisms. *Mol Cell Biol*, **20**, 530-541.
49. Cross, J.C., Baczyk, D., Dobric, N., Hemberger, M., Hughes, M., Simmons, D.G., Yamamoto, H. and Kingdom, J.C. (2003) Genes, development and evolution of the placenta. *Placenta*, **24**, 123-130.
50. Hollenberg, S.M., Sternglanz, R., Cheng, P.F. and Weintraub, H. (1995) Identification of a new family of tissue-specific basic helix-loop-helix proteins with a two-hybrid system. *Mol Cell Biol*, **15**, 3813-3822.
51. Firulli, B.A., Fuchs, R.K., Vincentz, J.W., Clouthier, D.E. and Firulli, A.B. (2014) Hand1 phosphoregulation within the distal arch neural crest is essential for craniofacial morphogenesis. *Development*, **141**, 3050-3061.
52. Yamagishi, H., Olson, E.N. and Srivastava, D. (2000) The basic helix-loop-helix transcription factor, dHAND, is required for vascular development. *J Clin Invest*, **105**, 261-270.
53. Abe, M., Michikami, I., Fukushi, T., Abe, A., Maeda, Y., Ooshima, T. and Wakisaka, S. (2010) Hand2 regulates chondrogenesis in vitro and in vivo. *Bone*, **46**, 1359-1368.
54. Funato, N., Chapman, S.L., McKee, M.D., Funato, H., Morris, J.A., Shelton, J.M., Richardson, J.A. and Yanagisawa, H. (2009) Hand2 controls osteoblast differentiation in the branchial arch by inhibiting DNA binding of Runx2. *Development*, **136**, 615-625.
55. Natarajan, A., Yamagishi, H., Ahmad, F., Li, D., Roberts, R., Matsuoka, R., Hill, S. and Srivastava, D. (2001) Human eHAND, but not dHAND, is down-regulated in cardiomyopathies. *J Mol Cell Cardiol*, **33**, 1607-1614.
56. Tamura, M., Amano, T. and Shiroishi, T. (2014) The Hand2 gene dosage effect in developmental defects and human congenital disorders. *Curr Top Dev Biol*, **110**, 129-152.
57. Krapp, A., Knofler, M., Frutiger, S., Hughes, G.J., Hagenbuchle, O. and Wellauer, P.K. (1996) The p48 DNA-binding subunit of transcription factor PTF1 is a new exocrine pancreas-specific basic helix-loop-helix protein. *EMBO J*, **15**, 4317-4329.
58. Krapp, A., Knofler, M., Ledermann, B., Burki, K., Berney, C., Zoerkler, N., Hagenbuchle, O. and Wellauer, P.K. (1998) The bHLH protein PTF1-p48 is essential for the formation of the exocrine and the correct spatial organization of the endocrine pancreas. *Genes Dev*, **12**, 3752-3763.
59. Rose, S.D., Swift, G.H., Peyton, M.J., Hammer, R.E. and MacDonald, R.J. (2001) The role of PTF1-P48 in pancreatic acinar gene expression. *J Biol Chem*, **276**, 44018-44026.
60. Hori, K., Cholewa-Waclaw, J., Nakada, Y., Glasgow, S.M., Masui, T., Henke, R.M., Wildner, H., Martarelli, B., Beres, T.M., Epstein, J.A. *et al.* (2008) A nonclassical bHLH Rbpj transcription factor complex is required for specification of GABAergic neurons independent of Notch signaling. *Genes Dev*, **22**, 166-178.
61. Sellick, G.S., Barker, K.T., Stolte-Dijkstra, I., Fleischmann, C., Coleman, R.J., Garrett, C., Gloyn, A.L., Edghill, E.L., Hattersley, A.T., Wellauer, P.K. *et al.* (2004) Mutations in PTF1A cause pancreatic and cerebellar agenesis. *Nat Genet*, **36**, 1301-1305.
62. McCormick, M.B., Tamimi, R.M., Snider, L., Asakura, A., Bergstrom, D. and Tapscott, S.J. (1996) NeuroD2 and neuroD3: distinct expression patterns and transcriptional activation potentials within the neuroD gene family. *Mol Cell Biol*, **16**, 5792-5800.
63. Poulin, G., Turgeon, B. and Drouin, J. (1997) NeuroD1/beta2 contributes to cell-specific transcription of the proopiomelanocortin gene. *Mol Cell Biol*, **17**, 6673-6682.
64. Sharma, A., Moore, M., Marcora, E., Lee, J.E., Qiu, Y., Samaras, S. and Stein, R. (1999) The NeuroD1/BETA2 sequences essential for insulin gene transcription colocalize with those necessary for neurogenesis and p300/CREB binding protein binding. *Mol Cell Biol*, **19**, 704-713.

65. Farah, M.H., Olson, J.M., Sucic, H.B., Hume, R.I., Tapscott, S.J. and Turner, D.L. (2000) Generation of neurons by transient expression of neural bHLH proteins in mammalian cells. *Development*, **127**, 693-702.
66. Franklin, A., Kao, A., Tapscott, S. and Unis, A. (2001) NeuroD homologue expression during cortical development in the human brain. *J Child Neurol*, **16**, 849-853.
67. Westerman, B.A., Chhatta, A., Poutsma, A., van Vegchel, T. and Oudejans, C.B. (2004) NEUROD1 acts in vitro as an upstream regulator of NEUROD2 in trophoblast cells. *Biochim Biophys Acta*, **1676**, 96-103.
68. Ma, Q., Kintner, C. and Anderson, D.J. (1996) Identification of neurogenin, a vertebrate neuronal determination gene. *Cell*, **87**, 43-52.
69. Ma, Q., Anderson, D.J. and Fritzsch, B. (2000) Neurogenin 1 null mutant ears develop fewer, morphologically normal hair cells in smaller sensory epithelia devoid of innervation. *J Assoc Res Otolaryngol*, **1**, 129-143.
70. Sun, Y., Nadal-Vicens, M., Misono, S., Lin, M.Z., Zubiaga, A., Hua, X., Fan, G. and Greenberg, M.E. (2001) Neurogenin promotes neurogenesis and inhibits glial differentiation by independent mechanisms. *Cell*, **104**, 365-376.
71. Yang, Z., MacQuarrie, K.L., Analau, E., Tyler, A.E., Dilworth, F.J., Cao, Y., Diede, S.J. and Tapscott, S.J. (2009) MyoD and E-protein heterodimers switch rhabdomyosarcoma cells from an arrested myoblast phase to a differentiated state. *Genes Dev*, **23**, 694-707.
72. Huang, C., Chan, J.A. and Schuurmans, C. (2014) Proneural bHLH genes in development and disease. *Curr Top Dev Biol*, **110**, 75-127.
73. Galvez, H., Tena, J.J., Giraldez, F. and Abello, G. (2017) The Repression of Atoh1 by Neurogenin1 during Inner Ear Development. *Front Mol Neurosci*, **10**, 321.
74. Roztocil, T., Matter-Sadzinski, L., Alliod, C., Ballivet, M. and Matter, J.M. (1997) NeuroM, a neural helix-loop-helix transcription factor, defines a new transition stage in neurogenesis. *Development*, **124**, 3263-3272.
75. Takebayashi, K., Takahashi, S., Yokota, C., Tsuda, H., Nakanishi, S., Asashima, M. and Kageyama, R. (1997) Conversion of ectoderm into a neural fate by ATH-3, a vertebrate basic helix-loop-helix gene homologous to Drosophila proneural gene atonal. *EMBO J*, **16**, 384-395.
76. Shimizu, C., Akazawa, C., Nakanishi, S. and Kageyama, R. (1995) MATH-2, a mammalian helix-loop-helix factor structurally related to the product of Drosophila proneural gene atonal, is specifically expressed in the nervous system. *Eur J Biochem*, **229**, 239-248.
77. Schwab, M.H., Bartholomae, A., Heimrich, B., Feldmeyer, D., Druffel-Augustin, S., Goebbels, S., Naya, F.J., Zhao, S., Frotscher, M., Tsai, M.J. *et al.* (2000) Neuronal basic helix-loop-helix proteins (NEX and BETA2/Neuro D) regulate terminal granule cell differentiation in the hippocampus. *J Neurosci*, **20**, 3714-3724.
78. Nakada, Y., Hunsaker, T.L., Henke, R.M. and Johnson, J.E. (2004) Distinct domains within Mash1 and Math1 are required for function in neuronal differentiation versus neuronal cell-type specification. *Development*, **131**, 1319-1330.
79. Bermingham, N.A., Hassan, B.A., Price, S.D., Vollrath, M.A., Ben-Arie, N., Eatock, R.A., Bellen, H.J., Lysakowski, A. and Zoghbi, H.Y. (1999) Math1: an essential gene for the generation of inner ear hair cells. *Science*, **284**, 1837-1841.
80. Akazawa, C., Ishibashi, M., Shimizu, C., Nakanishi, S. and Kageyama, R. (1995) A mammalian helix-loop-helix factor structurally related to the product of Drosophila proneural gene atonal is a positive transcriptional regulator expressed in the developing nervous system. *J Biol Chem*, **270**, 8730-8738.
81. Yang, Q., Bermingham, N.A., Finegold, M.J. and Zoghbi, H.Y. (2001) Requirement of Math1 for secretory cell lineage commitment in the mouse intestine. *Science*, **294**, 2155-2158.
82. Gradwohl, G., Fode, C. and Guillemot, F. (1996) Restricted expression of a novel murine atonal-related bHLH protein in undifferentiated neural precursors. *Dev Biol*, **180**, 227-241.
83. Fode, C., Gradwohl, G., Morin, X., Dierich, A., LeMeur, M., Goridis, C. and Guillemot, F. (1998) The bHLH protein NEUROGENIN 2 is a determination factor for epibranchial placode-derived sensory neurons. *Neuron*, **20**, 483-494.
84. Rukstalis, J.M. and Habener, J.F. (2009) Neurogenin3: a master regulator of pancreatic islet differentiation and regeneration. *Islets*, **1**, 177-184.
85. Roark, R., Itzhaki, L. and Philpott, A. (2012) Complex regulation controls Neurogenin3 proteolysis. *Biol Open*, **1**, 1264-1272.
86. Pelling, M., Anthwal, N., McNay, D., Gradwohl, G., Leiter, A.B., Guillemot, F. and Ang, S.L. (2011) Differential requirements for neurogenin 3 in the development of POMC and NPY neurons in the hypothalamus. *Dev Biol*, **349**, 406-416.

87. Brown, N.L., Kanekar, S., Vetter, M.L., Tucker, P.K., Gemza, D.L. and Glaser, T. (1998) Math5 encodes a murine basic helix-loop-helix transcription factor expressed during early stages of retinal neurogenesis. *Development*, **125**, 4821-4833.
88. Saul, S.M., Brzezinski, J.A.t., Altschuler, R.A., Shore, S.E., Rudolph, D.D., Kabara, L.L., Halsey, K.E., Hufnagel, R.B., Zhou, J., Dolan, D.F. *et al.* (2008) Math5 expression and function in the central auditory system. *Mol Cell Neurosci*, **37**, 153-169.
89. Inoue, C., Bae, S.K., Takatsuka, K., Inoue, T., Bessho, Y. and Kageyama, R. (2001) Math6, a bHLH gene expressed in the developing nervous system, regulates neuronal versus glial differentiation. *Genes Cells*, **6**, 977-986.
90. Ross, M.D., Martinka, S., Mukherjee, A., Sedor, J.R., Vinson, C. and Bruggeman, L.A. (2006) Math6 expression during kidney development and altered expression in a mouse model of glomerulosclerosis. *Dev Dyn*, **235**, 3102-3109.
91. Lynn, F.C., Sanchez, L., Gomis, R., German, M.S. and Gasa, R. (2008) Identification of the bHLH factor Math6 as a novel component of the embryonic pancreas transcriptional network. *PLoS One*, **3**, e2430.
92. Yao, J., Zhou, J., Liu, Q., Lu, D., Wang, L., Qiao, X. and Jia, W. (2010) Atoh8, a bHLH transcription factor, is required for the development of retina and skeletal muscle in zebrafish. *PLoS One*, **5**, e10945.
93. Schroeder, N., Wuelling, M., Hoffmann, D., Brand-Saberi, B. and Vortkamp, A. (2019) Atoh8 acts as a regulator of chondrocyte proliferation and differentiation in endochondral bones. *PLoS One*, **14**, e0218230.
94. Fang, F., Wasserman, S.M., Torres-Vazquez, J., Weinstein, B., Cao, F., Li, Z., Wilson, K.D., Yue, W., Wu, J.C., Xie, X. *et al.* (2014) The role of Hath6, a newly identified shear-stress-responsive transcription factor, in endothelial cell differentiation and function. *J Cell Sci*, **127**, 1428-1440.
95. Patel, N., Varghese, J., Masaratana, P., Latunde-Dada, G.O., Jacob, M., Simpson, R.J. and McKie, A.T. (2014) The transcription factor ATOH8 is regulated by erythropoietic activity and regulates HAMP transcription and cellular pSMAD1,5,8 levels. *Br J Haematol*, **164**, 586-596.
96. Zhu, L., Tran, T., Rukstalis, J.M., Sun, P., Damsz, B. and Konieczny, S.F. (2004) Inhibition of Mist1 homodimer formation induces pancreatic acinar-to-ductal metaplasia. *Mol Cell Biol*, **24**, 2673-2681.
97. Tran, T., Jia, D., Sun, Y. and Konieczny, S.F. (2007) The bHLH domain of Mistl is sufficient to activate gene transcription. *Gene Expr*, **13**, 241-253.
98. Lemercier, C., To, R.Q., Swanson, B.J., Lyons, G.E. and Konieczny, S.F. (1997) Mist1: a novel basic helix-loop-helix transcription factor exhibits a developmentally regulated expression pattern. *Dev Biol*, **182**, 101-113.
99. McLellan, A.S., Langlands, K. and Kealey, T. (2002) Exhaustive identification of human class II basic helix-loop-helix proteins by virtual library screening. *Mech Dev*, **119 Suppl 1**, S285-291.
100. Guillemot, F. and Joyner, A.L. (1993) Dynamic expression of the murine Achaete-Scute homologue Mash-1 in the developing nervous system. *Mech Dev*, **42**, 171-185.
101. Wang, C.Y., Shahi, P., Huang, J.T., Phan, N.N., Sun, Z., Lin, Y.C., Lai, M.D. and Werb, Z. (2017) Systematic analysis of the achaete-scute complex-like gene signature in clinical cancer patients. *Mol Clin Oncol*, **6**, 7-18.
102. Johnson, J.E., Birren, S.J. and Anderson, D.J. (1990) Two rat homologues of Drosophila achaete-scute specifically expressed in neuronal precursors. *Nature*, **346**, 858-861.
103. Liu, X., Chen, X., Zhong, B., Wang, A., Wang, X., Chu, F., Nurieva, R.I., Yan, X., Chen, P., van der Flier, L.G. *et al.* (2014) Transcription factor achaete-scute homologue 2 initiates follicular T-helper-cell development. *Nature*, **507**, 513-518.
104. Yoshida, S., Ohbo, K., Takakura, A., Takebayashi, H., Okada, T., Abe, K. and Nabeshima, Y. (2001) Sgn1, a basic helix-loop-helix transcription factor delineates the salivary gland duct cell lineage in mice. *Dev Biol*, **240**, 517-530.
105. Bullard, T., Koek, L., Roztocil, E., Kingsley, P.D., Mirels, L. and Ovitt, C.E. (2008) Ascl3 expression marks a progenitor population of both acinar and ductal cells in mouse salivary glands. *Dev Biol*, **320**, 72-78.
106. Weng, P.L., Vinjamuri, M. and Ovitt, C.E. (2016) Ascl3 transcription factor marks a distinct progenitor lineage for non-neuronal support cells in the olfactory epithelium. *Sci Rep*, **6**, 38199.
107. Jonsson, M., Bjorntorp Mark, E., Brantsing, C., Brandner, J.M., Lindahl, A. and Asp, J. (2004) Hash4, a novel human achaete-scute homologue found in fetal skin. *Genomics*, **84**, 859-866.
108. Kallianpur, A.R., Jordan, J.E. and Brandt, S.J. (1994) The SCL/TAL-1 gene is expressed in progenitors of both the hematopoietic and vascular systems during embryogenesis. *Blood*, **83**, 1200-1208.
109. Pulford, K., Lecointe, N., Leroy-Viard, K., Jones, M., Mathieu-Mahul, D. and Mason, D.Y. (1995) Expression of TAL-1 proteins in human tissues. *Blood*, **85**, 675-684.

110. Porcher, C., Swat, W., Rockwell, K., Fujiwara, Y., Alt, F.W. and Orkin, S.H. (1996) The T cell leukemia oncoprotein SCL/tal-1 is essential for development of all hematopoietic lineages. *Cell*, **86**, 47-57.
111. Drake, C.J., Brandt, S.J., Trusk, T.C. and Little, C.D. (1997) TAL1/SCL is expressed in endothelial progenitor cells/angioblasts and defines a dorsal-to-ventral gradient of vasculogenesis. *Dev Biol*, **192**, 17-30.
112. Achim, K., Peltopuro, P., Lahti, L., Tsai, H.H., Zachariah, A., Astrand, M., Salminen, M., Rowitch, D. and Partanen, J. (2013) The role of Tal2 and Tal1 in the differentiation of midbrain GABAergic neuron precursors. *Biol Open*, **2**, 990-997.
113. Mori, S., Sugawara, S., Kikuchi, T., Tanji, M., Narumi, O., Stoykova, A., Nishikawa, S.I. and Yokota, Y. (1999) The leukemic oncogene tal-2 is expressed in the developing mouse brain. *Brain Res Mol Brain Res*, **64**, 199-210.
114. Bucher, K., Sofroniew, M.V., Pannell, R., Impey, H., Smith, A.J., Torres, E.M., Dunnett, S.B., Jin, Y., Baer, R. and Rabbitts, T.H. (2000) The T cell oncogene Tal2 is necessary for normal development of the mouse brain. *Dev Biol*, **227**, 533-544.
115. Capron, C., Lecluse, Y., Kaushik, A.L., Foudi, A., Lacout, C., Sekkai, D., Godin, I., Albagli, O., Poullion, I., Svinartchouk, F. *et al.* (2006) The SCL relative LYL-1 is required for fetal and adult hematopoietic stem cell function and B-cell differentiation. *Blood*, **107**, 4678-4686.
116. Giroux, S., Kaushik, A.L., Capron, C., Jalil, A., Kelaidi, C., Sablitzky, F., Dumenil, D., Albagli, O. and Godin, I. (2007) lyl-1 and tal-1/scl, two genes encoding closely related bHLH transcription factors, display highly overlapping expression patterns during cardiovascular and hematopoietic ontogeny. *Gene Expr Patterns*, **7**, 215-226.
117. Begley, C.G., Lipkowitz, S., Gobel, V., Mahon, K.A., Bertness, V., Green, A.R., Gough, N.M. and Kirsch, I.R. (1992) Molecular characterization of NSCL, a gene encoding a helix-loop-helix protein expressed in the developing nervous system. *Proc Natl Acad Sci U S A*, **89**, 38-42.
118. Kruger, M. and Braun, T. (2002) The neuronal basic helix-loop-helix transcription factor NSCL-1 is dispensable for normal neuronal development. *Mol Cell Biol*, **22**, 792-800.
119. Cogliati, T., Good, D.J., Haigney, M., Delgado-Romero, P., Eckhaus, M.A., Koch, W.J. and Kirsch, I.R. (2002) Predisposition to arrhythmia and autonomic dysfunction in Nhlh1-deficient mice. *Mol Cell Biol*, **22**, 4977-4983.
120. Brown, L., Espinosa, R., 3rd, Le Beau, M.M., Siciliano, M.J. and Baer, R. (1992) HEN1 and HEN2: a subgroup of basic helix-loop-helix genes that are coexpressed in a human neuroblastoma. *Proc Natl Acad Sci U S A*, **89**, 8492-8496.
121. Gobel, V., Lipkowitz, S., Kozak, C.A. and Kirsch, I.R. (1992) NSCL-2: a basic domain helix-loop-helix gene expressed in early neurogenesis. *Cell Growth Differ*, **3**, 143-148.
122. Lu, J., Webb, R., Richardson, J.A. and Olson, E.N. (1999) MyoR: a muscle-restricted basic helix-loop-helix transcription factor that antagonizes the actions of MyoD. *Proc Natl Acad Sci U S A*, **96**, 552-557.
123. Acharya, A., Baek, S.T., Huang, G., Eskiocak, B., Goetsch, S., Sung, C.Y., Banfi, S., Sauer, M.F., Olsen, G.S., Duffield, J.S. *et al.* (2012) The bHLH transcription factor Tcf21 is required for lineage-specific EMT of cardiac fibroblast progenitors. *Development*, **139**, 2139-2149.
124. Hidai, H., Bardales, R., Goodwin, R., Quertermous, T. and Quertermous, E.E. (1998) Cloning of capsulin, a basic helix-loop-helix factor expressed in progenitor cells of the pericardium and the coronary arteries. *Mech Dev*, **73**, 33-43.
125. Quaggin, S.E., Vanden Heuvel, G.B. and Igarashi, P. (1998) Pod-1, a mesoderm-specific basic-helix-loop-helix protein expressed in mesenchymal and glomerular epithelial cells in the developing kidney. *Mech Dev*, **71**, 37-48.
126. Quaggin, S.E., Schwartz, L., Cui, S., Igarashi, P., Deimling, J., Post, M. and Rossant, J. (1999) The basic-helix-loop-helix protein pod1 is critically important for kidney and lung organogenesis. *Development*, **126**, 5771-5783.
127. Robb, L., Mifsud, L., Hartley, L., Biben, C., Copeland, N.G., Gilbert, D.J., Jenkins, N.A. and Harvey, R.P. (1998) epicardin: A novel basic helix-loop-helix transcription factor gene expressed in epicardium, branchial arch myoblasts, and mesenchyme of developing lung, gut, kidney, and gonads. *Dev Dyn*, **213**, 105-113.
128. Narumi, O., Mori, S., Boku, S., Tsuji, Y., Hashimoto, N., Nishikawa, S. and Yokota, Y. (2000) OUT, a novel basic helix-loop-helix transcription factor with an Id-like inhibitory activity. *J Biol Chem*, **275**, 3510-3521.
129. Kommagani, R., Szwarc, M.M., Kovanci, E., Creighton, C.J., O'Malley, B.W., Demayo, F.J. and Lydon, J.P. (2014) A murine uterine transcriptome, responsive to steroid receptor coactivator-2, reveals transcription factor 23 as essential for decidualization of human endometrial stromal cells. *Biol Reprod*, **90**, 75.
130. Klopocki, E., Lohan, S., Doelken, S.C., Stricker, S., Ockeloen, C.W., Soares Thiele de Aguiar, R., Lezirovitz, K., Mingroni Netto, R.C., Jamsheer, A., Shah, H. *et al.* (2012) Duplications of BHLHA9 are associated with ectrodactyly and tibia hemimelia inherited in non-Mendelian fashion. *J Med Genet*, **49**, 119-125.

131. Malik, S., Percin, F.E., Bornholdt, D., Albrecht, B., Percesepe, A., Koch, M.C., Landi, A., Fritz, B., Khan, R., Mumtaz, S. *et al.* (2014) Mutations affecting the BHLHA9 DNA-binding domain cause MSSD, mesoaxial synostotic syndactyly with phalangeal reduction, Malik-Percin type. *Am J Hum Genet*, **95**, 649-659.
132. Schatz, O., Langer, E. and Ben-Arie, N. (2014) Gene dosage of the transcription factor Fingerin (bHLHA9) affects digit development and links syndactyly to ectrodactyly. *Hum Mol Genet*, **23**, 5394-5401.
133. Feng, L., Xie, X., Joshi, P.S., Yang, Z., Shibasaki, K., Chow, R.L. and Gan, L. (2006) Requirement for Bhlhb5 in the specification of amacrine and cone bipolar subtypes in mouse retina. *Development*, **133**, 4815-4825.
134. Xu, Z.P., Dutra, A., Stellrecht, C.M., Wu, C., Piatigorsky, J. and Saunders, G.F. (2002) Functional and structural characterization of the human gene BHLHB5, encoding a basic helix-loop-helix transcription factor. *Genomics*, **80**, 311-318.
135. Skaggs, K., Martin, D.M. and Novitch, B.G. (2011) Regulation of spinal interneuron development by the Olig-related protein Bhlhb5 and Notch signaling. *Development*, **138**, 3199-3211.
136. Kim, M.H., Gunnersen, J., Augustine, C. and Tan, S.S. (2002) Region-specific expression of the helix-loop-helix gene BETA3 in developing and adult brains. *Mech Dev*, **114**, 125-128.
137. Bramblett, D.E., Copeland, N.G., Jenkins, N.A. and Tsai, M.J. (2002) BHLHB4 is a bHLH transcriptional regulator in pancreas and brain that marks the dimesencephalic boundary. *Genomics*, **79**, 402-412.
138. Bramblett, D.E., Pennesi, M.E., Wu, S.M. and Tsai, M.J. (2004) The transcription factor Bhlhb4 is required for rod bipolar cell maturation. *Neuron*, **43**, 779-793.
139. Dai, J., Bercury, K.K., Ahrendsen, J.T. and Macklin, W.B. (2015) Olig1 function is required for oligodendrocyte differentiation in the mouse brain. *J Neurosci*, **35**, 4386-4402.
140. Novitch, B.G., Chen, A.I. and Jessell, T.M. (2001) Coordinate regulation of motor neuron subtype identity and pan-neuronal properties by the bHLH repressor Olig2. *Neuron*, **31**, 773-789.
141. Zhou, Q., Choi, G. and Anderson, D.J. (2001) The bHLH transcription factor Olig2 promotes oligodendrocyte differentiation in collaboration with Nkx2.2. *Neuron*, **31**, 791-807.
142. Storm, R., Cholewa-Waclaw, J., Reuter, K., Brohl, D., Sieber, M., Treier, M., Muller, T. and Birchmeier, C. (2009) The bHLH transcription factor Olig3 marks the dorsal neuroepithelium of the hindbrain and is essential for the development of brainstem nuclei. *Development*, **136**, 295-305.
143. Muller, T., Anlag, K., Wildner, H., Britsch, S., Treier, M. and Birchmeier, C. (2005) The bHLH factor Olig3 coordinates the specification of dorsal neurons in the spinal cord. *Genes Dev*, **19**, 733-743.
144. Boudjelal, M., Taneja, R., Matsubara, S., Bouillet, P., Dolle, P. and Chambon, P. (1997) Overexpression of Stra13, a novel retinoic acid-inducible gene of the basic helix-loop-helix family, inhibits mesodermal and promotes neuronal differentiation of P19 cells. *Genes Dev*, **11**, 2052-2065.
145. Ow, J.R., Tan, Y.H., Jin, Y., Bahirvani, A.G. and Taneja, R. (2014) Stra13 and Sharp-1, the non-grouchy regulators of development and disease. *Curr Top Dev Biol*, **110**, 317-338.
146. Meinhardt, G., Husslein, P. and Knofler, M. (2005) Tissue-specific and ubiquitous basic helix-loop-helix transcription factors in human placental trophoblasts. *Placenta*, **26**, 527-539.
147. Azmi, S., Ozog, A. and Taneja, R. (2004) Sharp-1/DEC2 inhibits skeletal muscle differentiation through repression of myogenic transcription factors. *J Biol Chem*, **279**, 52643-52652.
148. Gulbagci, N.T., Li, L., Ling, B., Gopinadhan, S., Walsh, M., Rossner, M., Nave, K.A. and Taneja, R. (2009) SHARP1/DEC2 inhibits adipogenic differentiation by regulating the activity of C/EBP. *EMBO Rep*, **10**, 79-86.
149. Lyden, D., Young, A.Z., Zagzag, D., Yan, W., Gerald, W., O'Reilly, R., Bader, B.L., Hynes, R.O., Zhuang, Y., Manova, K. *et al.* (1999) Id1 and Id3 are required for neurogenesis, angiogenesis and vascularization of tumour xenografts. *Nature*, **401**, 670-677.
150. Bai, G., Sheng, N., Xie, Z., Bian, W., Yokota, Y., Benezra, R., Kageyama, R., Guillemot, F. and Jing, N. (2007) Id sustains Hes1 expression to inhibit precocious neurogenesis by releasing negative autoregulation of Hes1. *Dev Cell*, **13**, 283-297.
151. Murre, C. (2019) Helix-loop-helix proteins and the advent of cellular diversity: 30 years of discovery. *Genes Dev*, **33**, 6-25.
152. Riechmann, V., van Cruchten, I. and Sablitzky, F. (1994) The expression pattern of Id4, a novel dominant negative helix-loop-helix protein, is distinct from Id1, Id2 and Id3. *Nucleic Acids Res*, **22**, 749-755.
153. Moldes, M., Boizard, M., Liepvre, X.L., Feve, B., Dugail, I. and Pairault, J. (1999) Functional antagonism between inhibitor of DNA binding (Id) and adipocyte determination and differentiation factor 1/sterol regulatory element-

binding protein-1c (ADD1/SREBP-1c) trans-factors for the regulation of fatty acid synthase promoter in adipocytes. *Biochem J*, **344 Pt 3**, 873-880.
154. Yokota, Y., Mansouri, A., Mori, S., Sugawara, S., Adachi, S., Nishikawa, S. and Gruss, P. (1999) Development of peripheral lymphoid organs and natural killer cells depends on the helix-loop-helix inhibitor Id2. *Nature*, **397**, 702-706.
155. Pan, L., Sato, S., Frederick, J.P., Sun, X.H. and Zhuang, Y. (1999) Impaired immune responses and B-cell proliferation in mice lacking the Id3 gene. *Mol Cell Biol*, **19**, 5969-5980.
156. Tokuzawa, Y., Yagi, K., Yamashita, Y., Nakachi, Y., Nikaido, I., Bono, H., Ninomiya, Y., Kanesaki-Yatsuka, Y., Akita, M., Motegi, H. *et al.* (2010) Id4, a new candidate gene for senile osteoporosis, acts as a molecular switch promoting osteoblast differentiation. *PLoS Genet*, **6**, e1001019.
157. Murad, J.M., Place, C.S., Ran, C., Hekmatyar, S.K., Watson, N.P., Kauppinen, R.A. and Israel, M.A. (2010) Inhibitor of DNA binding 4 (ID4) regulation of adipocyte differentiation and adipose tissue formation in mice. *J Biol Chem*, **285**, 24164-24173.
158. Fischer, A., Schumacher, N., Maier, M., Sendtner, M. and Gessler, M. (2004) The Notch target genes Hey1 and Hey2 are required for embryonic vascular development. *Genes Dev*, **18**, 901-911.
159. Weber, D., Wiese, C. and Gessler, M. (2014) Hey bHLH transcription factors. *Curr Top Dev Biol*, **110**, 285-315.
160. Chin, M.T., Maemura, K., Fukumoto, S., Jain, M.K., Layne, M.D., Watanabe, M., Hsieh, C.M. and Lee, M.E. (2000) Cardiovascular basic helix loop helix factor 1, a novel transcriptional repressor expressed preferentially in the developing and adult cardiovascular system. *J Biol Chem*, **275**, 6381-6387.
161. Noguchi, Y.T., Nakamura, M., Hino, N., Nogami, J., Tsuji, S., Sato, T., Zhang, L., Tsujikawa, K., Tanaka, T., Izawa, K. *et al.* (2019) Cell-autonomous and redundant roles of Hey1 and HeyL in muscle stem cells: HeyL requires Hes1 to bind diverse DNA sites. *Development*, **146**.
162. Ishibashi, M., Ang, S.L., Shiota, K., Nakanishi, S., Kageyama, R. and Guillemot, F. (1995) Targeted disruption of mammalian hairy and Enhancer of split homolog-1 (HES-1) leads to up-regulation of neural helix-loop-helix factors, premature neurogenesis, and severe neural tube defects. *Genes Dev*, **9**, 3136-3148.
163. Ju, B.G., Solum, D., Song, E.J., Lee, K.J., Rose, D.W., Glass, C.K. and Rosenfeld, M.G. (2004) Activating the PARP-1 sensor component of the groucho/ TLE1 corepressor complex mediates a CaMKinase IIdelta-dependent neurogenic gene activation pathway. *Cell*, **119**, 815-829.
164. Ross, D.A., Hannenhalli, S., Tobias, J.W., Cooch, N., Shiekhattar, R. and Kadesch, T. (2006) Functional analysis of Hes-1 in preadipocytes. *Mol Endocrinol*, **20**, 698-705.
165. Kawamata, S., Du, C., Li, K. and Lavau, C. (2002) Overexpression of the Notch target genes Hes in vivo induces lymphoid and myeloid alterations. *Oncogene*, **21**, 3855-3863.
166. Kageyama, R., Ohtsuka, T. and Kobayashi, T. (2007) The Hes gene family: repressors and oscillators that orchestrate embryogenesis. *Development*, **134**, 1243-1251.
167. Sang, L. and Coller, H.A. (2009) Fear of commitment: Hes1 protects quiescent fibroblasts from irreversible cellular fates. *Cell Cycle*, **8**, 2161-2167.
168. Kobayashi, T. and Kageyama, R. (2014) Expression dynamics and functions of Hes factors in development and diseases. *Curr Top Dev Biol*, **110**, 263-283.
169. Solter, M., Locker, M., Boy, S., Taelman, V., Bellefroid, E.J., Perron, M. and Pieler, T. (2006) Characterization and function of the bHLH-O protein XHes2: insight into the mechanisms controlling retinal cell fate decision. *Development*, **133**, 4097-4108.
170. Akazawa, C., Sasai, Y., Nakanishi, S. and Kageyama, R. (1992) Molecular characterization of a rat negative regulator with a basic helix-loop-helix structure predominantly expressed in the developing nervous system. *J Biol Chem*, **267**, 21879-21885.
171. Maier, E. and Gunhaga, L. (2009) Dynamic expression of neurogenic markers in the developing chick olfactory epithelium. *Dev Dyn*, **238**, 1617-1625.
172. Gao, X., Chandra, T., Gratton, M.O., Quelo, I., Prud'homme, J., Stifani, S. and St-Arnaud, R. (2001) HES6 acts as a transcriptional repressor in myoblasts and can induce the myogenic differentiation program. *J Cell Biol*, **154**, 1161-1171.
173. Cossins, J., Vernon, A.E., Zhang, Y., Philpott, A. and Jones, P.H. (2002) Hes6 regulates myogenic differentiation. *Development*, **129**, 2195-2207.
174. Vasiliauskas, D. and Stern, C.D. (2000) Expression of mouse HES-6, a new member of the Hairy/Enhancer of split family of bHLH transcription factors. *Mech Dev*, **98**, 133-137.

175. Koyano-Nakagawa, N., Kim, J., Anderson, D. and Kintner, C. (2000) Hes6 acts in a positive feedback loop with the neurogenins to promote neuronal differentiation. *Development*, **127**, 4203-4216.
176. Jhas, S., Ciura, S., Belanger-Jasmin, S., Dong, Z., Llamosas, E., Theriault, F.M., Joachim, K., Tang, Y., Liu, L., Liu, J. *et al.* (2006) Hes6 inhibits astrocyte differentiation and promotes neurogenesis through different mechanisms. *J Neurosci*, **26**, 11061-11071.
177. Bessho, Y., Miyoshi, G., Sakata, R. and Kageyama, R. (2001) Hes7: a bHLH-type repressor gene regulated by Notch and expressed in the presomitic mesoderm. *Genes Cells*, **6**, 175-185.
178. Bessho, Y., Hirata, H., Masamizu, Y. and Kageyama, R. (2003) Periodic repression by the bHLH factor Hes7 is an essential mechanism for the somite segmentation clock. *Genes Dev*, **17**, 1451-1456.
179. Nakatani, T., Mizuhara, E., Minaki, Y., Sakamoto, Y. and Ono, Y. (2004) Helt, a novel basic-helix-loop-helix transcriptional repressor expressed in the developing central nervous system. *J Biol Chem*, **279**, 16356-16367.
180. Wende, C.Z., Zoubaa, S., Blak, A., Echevarria, D., Martinez, S., Guillemot, F., Wurst, W. and Guimera, J. (2015) Hairy/Enhancer-of-Split MEGANE and Proneural MASH1 Factors Cooperate Synergistically in Midbrain GABAergic Neurogenesis. *PLoS One*, **10**, e0127681.
181. Lluis, F., Ballestar, E., Suelves, M., Esteller, M. and Munoz-Canoves, P. (2005) E47 phosphorylation by p38 MAPK promotes MyoD/E47 association and muscle-specific gene transcription. *EMBO J*, **24**, 974-984.
182. Johnson, S.E., Wang, X., Hardy, S., Taparowsky, E.J. and Konieczny, S.F. (1996) Casein kinase II increases the transcriptional activities of MRF4 and MyoD independently of their direct phosphorylation. *Mol Cell Biol*, **16**, 1604-1613.
183. Teachenor, R., Beck, K., Wright, L.Y., Shen, Z., Briggs, S.P. and Murre, C. (2012) Biochemical and phosphoproteomic analysis of the helix-loop-helix protein E47. *Mol Cell Biol*, **32**, 1671-1682.
184. Hardwick, L.J.A., Davies, J.D. and Philpott, A. (2019) Multi-site phosphorylation controls the neurogenic and myogenic activity of E47. *Biochem Biophys Res Commun*, **511**, 111-116.
185. Lassar, A.B., Davis, R.L., Wright, W.E., Kadesch, T., Murre, C., Voronova, A., Baltimore, D. and Weintraub, H. (1991) Functional activity of myogenic HLH proteins requires hetero-oligomerization with E12/E47-like proteins in vivo. *Cell*, **66**, 305-315.
186. Bagchi, R.A., Wang, R., Jahan, F., Wigle, J.T. and Czubryt, M.P. (2016) Regulation of scleraxis transcriptional activity by serine phosphorylation. *J Mol Cell Cardiol*, **92**, 140-148.
187. Mammoto, T., Jiang, A., Jiang, E. and Mammoto, A. (2016) Role of Twist1 Phosphorylation in Angiogenesis and Pulmonary Fibrosis. *Am J Respir Cell Mol Biol*, **55**, 633-644.
188. Firulli, B.A., Redick, B.A., Conway, S.J. and Firulli, A.B. (2007) Mutations within helix I of Twist1 result in distinct limb defects and variation of DNA binding affinities. *J Biol Chem*, **282**, 27536-27546.
189. Firulli, A.B. (2003) A HANDful of questions: the molecular biology of the heart and neural crest derivatives (HAND)-subclass of basic helix-loop-helix transcription factors. *Gene*, **312**, 27-40.
190. Martindill, D.M., Risebro, C.A., Smart, N., Franco-Viseras Mdel, M., Rosario, C.O., Swallow, C.J., Dennis, J.W. and Riley, P.R. (2007) Nucleolar release of Hand1 acts as a molecular switch to determine cell fate. *Nat Cell Biol*, **9**, 1131-1141.
191. Cheng, Y.F., Tong, M. and Edge, A.S. (2016) Destabilization of Atoh1 by E3 Ubiquitin Ligase Huwe1 and Casein Kinase 1 Is Essential for Normal Sensory Hair Cell Development. *J Biol Chem*, **291**, 21096-21109.
192. Ma, Y.C., Song, M.R., Park, J.P., Henry Ho, H.Y., Hu, L., Kurtev, M.V., Zieg, J., Ma, Q., Pfaff, S.L. and Greenberg, M.E. (2008) Regulation of motor neuron specification by phosphorylation of neurogenin 2. *Neuron*, **58**, 65-77.
193. Li, S., Mattar, P., Zinyk, D., Singh, K., Chaturvedi, C.P., Kovach, C., Dixit, R., Kurrasch, D.M., Ma, Y.C., Chan, J.A. *et al.* (2012) GSK3 temporally regulates neurogenin 2 proneural activity in the neocortex. *J Neurosci*, **32**, 7791-7805.
194. Goldfarb, A.N. and Lewandowska, K. (1995) Inhibition of cellular differentiation by the SCL/tal oncoprotein: transcriptional repression by an Id-like mechanism. *Blood*, **85**, 465-471.
195. Deleuze, V., El-Hajj, R., Chalhoub, E., Dohet, C., Pinet, V., Couttet, P. and Mathieu, D. (2012) Angiopoietin-2 is a direct transcriptional target of TAL1, LYL1 and LMO2 in endothelial cells. *PLoS One*, **7**, e40484.
196. Xia, Y., Hwang, L.Y., Cobb, M.H. and Baer, R. (1994) Products of the TAL2 oncogene in leukemic T cells: bHLH phosphoproteins with DNA-binding activity. *Oncogene*, **9**, 1437-1446.
197. Brown, L. and Baer, R. (1994) HEN1 encodes a 20-kilodalton phosphoprotein that binds an extended E-box motif as a homodimer. *Mol Cell Biol*, **14**, 1245-1255.
198. Li, H., de Faria, J.P., Andrew, P., Nitarska, J. and Richardson, W.D. (2011) Phosphorylation regulates OLIG2 cofactor choice and the motor neuron-oligodendrocyte fate switch. *Neuron*, **69**, 918-929.

199. Hara, E., Hall, M. and Peters, G. (1997) Cdk2-dependent phosphorylation of Id2 modulates activity of E2A-related transcription factors. *EMBO J*, **16**, 332-342.
200. Deed, R.W., Hara, E., Atherton, G.T., Peters, G. and Norton, J.D. (1997) Regulation of Id3 cell cycle function by Cdk-2-dependent phosphorylation. *Mol Cell Biol*, **17**, 6815-6821.
201. Lopez-Mateo, I., Arruabarrena-Aristorena, A., Artaza-Irigaray, C., Lopez, J.A., Calvo, E. and Belandia, B. (2016) HEY1 functions are regulated by its phosphorylation at Ser-68. *Biosci Rep*, **36**.
202. Belanger-Jasmin, S., Llamosas, E., Tang, Y., Joachim, K., Osiceanu, A.M., Jhas, S. and Stifani, S. (2007) Inhibition of cortical astrocyte differentiation by Hes6 requires amino- and carboxy-terminal motifs important for dimerization and phosphorylation. *J Neurochem*, **103**, 2022-2034.
203. Gratton, M.O., Torban, E., Jasmin, S.B., Theriault, F.M., German, M.S. and Stifani, S. (2003) Hes6 promotes cortical neurogenesis and inhibits Hes1 transcription repression activity by multiple mechanisms. *Mol Cell Biol*, **23**, 6922-6935.
204. Sun, X.H. and Baltimore, D. (1991) An inhibitory domain of E12 transcription factor prevents DNA binding in E12 homodimers but not in E12 heterodimers. *Cell*, **64**, 459-470.
205. Dear, T.N., Hainzl, T., Follo, M., Nehls, M., Wilmore, H., Matena, K. and Boehm, T. (1997) Identification of interaction partners for the basic-helix-loop-helix protein E47. *Oncogene*, **14**, 891-898.
206. Murakami, M., Kataoka, K., Tominaga, J., Nakagawa, O. and Kurihara, H. (2004) Differential cooperation between dHAND and three different E-proteins. *Biochem Biophys Res Commun*, **323**, 168-174.
207. Hu, J.S., Olson, E.N. and Kingston, R.E. (1992) HEB, a helix-loop-helix protein related to E2A and ITF2 that can modulate the DNA-binding ability of myogenic regulatory factors. *Mol Cell Biol*, **12**, 1031-1042.
208. Langlands, K., Yin, X., Anand, G. and Prochownik, E.V. (1997) Differential interactions of Id proteins with basic-helix-loop-helix transcription factors. *J Biol Chem*, **272**, 19785-19793.
209. Benezra, R., Davis, R.L., Lockshon, D., Turner, D.L. and Weintraub, H. (1990) The protein Id: a negative regulator of helix-loop-helix DNA binding proteins. *Cell*, **61**, 49-59.
210. Lingbeck, J.M., Trausch-Azar, J.S., Ciechanover, A. and Schwartz, A.L. (2005) E12 and E47 modulate cellular localization and proteasome-mediated degradation of MyoD and Id1. *Oncogene*, **24**, 6376-6384.
211. Cubillo, E., Diaz-Lopez, A., Cuevas, E.P., Moreno-Bueno, G., Peinado, H., Montes, A., Santos, V., Portillo, F. and Cano, A. (2013) E47 and Id1 interplay in epithelial-mesenchymal transition. *PLoS One*, **8**, e59948.
212. Springhorn, J.P., Singh, K., Kelly, R.A. and Smith, T.W. (1994) Posttranscriptional regulation of Id1 activity in cardiac muscle. Alternative splicing of novel Id1 transcript permits homodimerization. *J Biol Chem*, **269**, 5132-5136.
213. Jen, Y., Weintraub, H. and Benezra, R. (1992) Overexpression of Id protein inhibits the muscle differentiation program: in vivo association of Id with E2A proteins. *Genes Dev*, **6**, 1466-1479.
214. Li, X., Wang, W., Wang, J., Malovannaya, A., Xi, Y., Li, W., Guerra, R., Hawke, D.H., Qin, J. and Chen, J. (2015) Proteomic analyses reveal distinct chromatin-associated and soluble transcription factor complexes. *Mol Syst Biol*, **11**, 775.
215. Massari, M.E., Rivera, R.R., Voland, J.R., Quong, M.W., Breit, T.M., van Dongen, J.J., de Smit, O. and Murre, C. (1998) Characterization of ABF-1, a novel basic helix-loop-helix transcription factor expressed in activated B lymphocytes. *Mol Cell Biol*, **18**, 3130-3139.
216. Jogi, A., Persson, P., Grynfeld, A., Pahlman, S. and Axelson, H. (2002) Modulation of basic helix-loop-helix transcription complex formation by Id proteins during neuronal differentiation. *J Biol Chem*, **277**, 9118-9126.
217. Loveys, D.A., Streiff, M.B. and Kato, G.J. (1996) E2A basic-helix-loop-helix transcription factors are negatively regulated by serum growth factors and by the Id3 protein. *Nucleic Acids Res*, **24**, 2813-2820.
218. Teo, Z., Chan, J.S.K., Chong, H.C., Sng, M.K., Choo, C.C., Phua, G.Z.M., Teo, D.J.R., Zhu, P., Choong, C., Wong, M.T.C. *et al.* (2017) Angiopoietin-like 4 induces a beta-catenin-mediated upregulation of ID3 in fibroblasts to reduce scar collagen expression. *Sci Rep*, **7**, 6303.
219. Leimeister, C., Dale, K., Fischer, A., Klamt, B., Hrabe de Angelis, M., Radtke, F., McGrew, M.J., Pourquie, O. and Gessler, M. (2000) Oscillating expression of c-Hey2 in the presomitic mesoderm suggests that the segmentation clock may use combinatorial signaling through multiple interacting bHLH factors. *Dev Biol*, **227**, 91-103.
220. Sasai, Y., Kageyama, R., Tagawa, Y., Shigemoto, R. and Nakanishi, S. (1992) Two mammalian helix-loop-helix factors structurally related to Drosophila hairy and Enhancer of split. *Genes Dev*, **6**, 2620-2634.
221. Sun, J., Kamei, C.N., Layne, M.D., Jain, M.K., Liao, J.K., Lee, M.E. and Chin, M.T. (2001) Regulation of myogenic terminal differentiation by the hairy-related transcription factor CHF2. *J Biol Chem*, **276**, 18591-18596.

222. Topno, N.S., Kannan, M. and Krishna, R. (2018) Mechanistic insights into the activity of Ptf1-p48 (pancreas transcription factor 1a): probing the interactions levels of Ptf1-p48 with E2A-E47 (transcription factor E2-alpha) and ID3 (inhibitor of DNA binding 3). *J Biomol Struct Dyn*, **36**, 1834-1852.
223. Firulli, B.A., Hadzic, D.B., McDaid, J.R. and Firulli, A.B. (2000) The basic helix-loop-helix transcription factors dHAND and eHAND exhibit dimerization characteristics that suggest complex regulation of function. *J Biol Chem*, **275**, 33567-33573.
224. Ghosh, B. and Leach, S.D. (2006) Interactions between hairy/enhancer of split-related proteins and the pancreatic transcription factor Ptf1-p48 modulate function of the PTF1 transcriptional complex. *Biochem J*, **393**, 679-685.
225. Taelman, V., Van Wayenbergh, R., Solter, M., Pichon, B., Pieler, T., Christophe, D. and Bellefroid, E.J. (2004) Sequences downstream of the bHLH domain of the Xenopus hairy-related transcription factor-1 act as an extended dimerization domain that contributes to the selection of the partners. *Dev Biol*, **276**, 47-63.
226. Samanta, J. and Kessler, J.A. (2004) Interactions between ID and OLIG proteins mediate the inhibitory effects of BMP4 on oligodendroglial differentiation. *Development*, **131**, 4131-4142.
227. Guo, S.J., Hu, J.G., Zhao, B.M., Shen, L., Wang, R., Zhou, J.S. and Lu, H.Z. (2011) Olig1 and ID4 interactions in living cells visualized by bimolecular fluorescence complementation technique. *Mol Biol Rep*, **38**, 4637-4642.
228. Isogai, E., Ohira, M., Ozaki, T., Oba, S., Nakamura, Y. and Nakagawara, A. (2011) Oncogenic LMO3 collaborates with HEN2 to enhance neuroblastoma cell growth through transactivation of Mash1. *PLoS One*, **6**, e19297.
229. Liu, A., Li, J., Marin-Husstege, M., Kageyama, R., Fan, Y., Gelinas, C. and Casaccia-Bonnefil, P. (2006) A molecular insight of Hes5-dependent inhibition of myelin gene expression: old partners and new players. *EMBO J*, **25**, 4833-4842.
230. Itoh, F., Itoh, S., Goumans, M.J., Valdimarsdottir, G., Iso, T., Dotto, G.P., Hamamori, Y., Kedes, L., Kato, M. and ten Dijke Pt, P. (2004) Synergy and antagonism between Notch and BMP receptor signaling pathways in endothelial cells. *EMBO J*, **23**, 541-551.
231. Salat, D., Winkler, A., Urlaub, H. and Gessler, M. (2015) Hey bHLH Proteins Interact with a FBXO45 Containing SCF Ubiquitin Ligase Complex and Induce Its Translocation into the Nucleus. *PLoS One*, **10**, e0130288.
232. Iso, T., Sartorelli, V., Poizat, C., Iezzi, S., Wu, H.Y., Chung, G., Kedes, L. and Hamamori, Y. (2001) HERP, a novel heterodimer partner of HES/E(spl) in Notch signaling. *Mol Cell Biol*, **21**, 6080-6089.
233. Bae, S., Bessho, Y., Hojo, M. and Kageyama, R. (2000) The bHLH gene Hes6, an inhibitor of Hes1, promotes neuronal differentiation. *Development*, **127**, 2933-2943.
234. Nakagawa, O., McFadden, D.G., Nakagawa, M., Yanagisawa, H., Hu, T., Srivastava, D. and Olson, E.N. (2000) Members of the HRT family of basic helix-loop-helix proteins act as transcriptional repressors downstream of Notch signaling. *Proc Natl Acad Sci U S A*, **97**, 13655-13660.
235. Coglievina, M., Guarnaccia, C., Pintar, A. and Pongor, S. (2010) Different degrees of structural order in distinct regions of the transcriptional repressor HES-1. *Biochim Biophys Acta*, **1804**, 2153-2161.
236. Ishibashi, M., Sasai, Y., Nakanishi, S. and Kageyama, R. (1993) Molecular characterization of HES-2, a mammalian helix-loop-helix factor structurally related to Drosophila hairy and Enhancer of split. *Eur J Biochem*, **215**, 645-652.
237. Hirata, H., Ohtsuka, T., Bessho, Y. and Kageyama, R. (2000) Generation of structurally and functionally distinct factors from the basic helix-loop-helix gene Hes3 by alternative first exons. *J Biol Chem*, **275**, 19083-19089.
238. De Obaldia, M.E., Bell, J.J., Wang, X., Harly, C., Yashiro-Ohtani, Y., DeLong, J.H., Zlotoff, D.A., Sultana, D.A., Pear, W.S. and Bhandoola, A. (2013) T cell development requires constraint of the myeloid regulator C/EBP-alpha by the Notch target and transcriptional repressor Hes1. *Nat Immunol*, **14**, 1277-1284.
239. Bessho, Y., Sakata, R., Komatsu, S., Shiota, K., Yamada, S. and Kageyama, R. (2001) Dynamic expression and essential functions of Hes7 in somite segmentation. *Genes Dev*, **15**, 2642-2647.
240. Iso, T., Sartorelli, V., Chung, G., Shichinohe, T., Kedes, L. and Hamamori, Y. (2001) HERP, a new primary target of Notch regulated by ligand binding. *Mol Cell Biol*, **21**, 6071-6079.
241. Iso, T., Kedes, L. and Hamamori, Y. (2003) HES and HERP families: multiple effectors of the Notch signaling pathway. *J Cell Physiol*, **194**, 237-255.
